# Identification of Novel Human 15-Lipoxygenase-2 (h15-LOX-2) Inhibitors Using a Virtual Screening Approach

**DOI:** 10.1021/acs.jmedchem.4c01884

**Published:** 2024-12-19

**Authors:** Lucas G. Viviani, Thais S. Iijima, Erika Piccirillo, Leandro Rezende, Thiago G. P. Alegria, Luis Eduardo S. Netto, Antonia T.-do Amaral, Sayuri Miyamoto

**Affiliations:** † Department of Biochemistry, Institute of Chemistry, University of São Paulo, Av. Prof. Lineu Prestes 748, 05508-000 São Paulo, Brazil; ‡ Center for Medicinal Chemistry (CQMED), State University of Campinas, Av. André Tosello 550, 13083-886 Campinas, Brazil; § Department of Genetics and Evolutionary Biology, Institute of Biosciences, University of São Paulo, Rua do Matão, 277, 05508-090 São Paulo, Brazil; ∥ Department of Fundamental Chemistry, Institute of Chemistry, University of São Paulo, Av. Prof. Lineu Prestes 748, 05508-000 São Paulo, Brazil

## Abstract

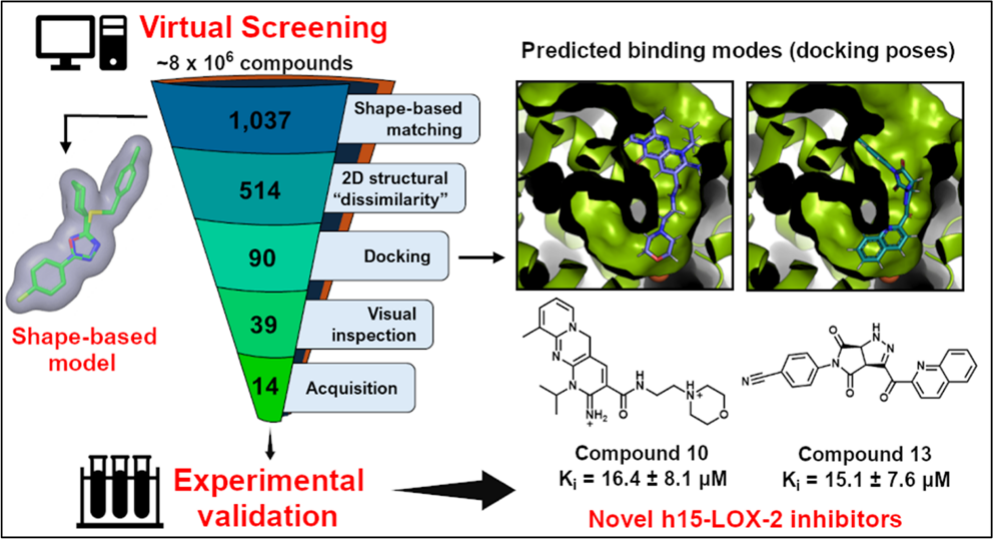

The human 15-lipoxygenase-2 (h15-LOX-2) catalyzes mainly the regio- and stereospecific oxygenation of arachidonate to its corresponding hydroperoxide (15(*S*)-HpETE). h15-LOX-2 is implicated in the biosynthesis of inflammatory lipid mediators and plays a role in the development of atherosclerotic plaques, but it is still underexploited as a drug target. Here, to search for novel h15-LOX-2 inhibitors, we used a virtual screening (VS) approach consisting of shape-based matching, two-dimensional (2D) structural “dissimilarity”, docking, and visual inspection filters, which were applied to a “curated” ZINC database (∼8 × 10^6^ compounds). The VS was experimentally validated, and six micromolar-range inhibitors were identified among 13 tested compounds (46.2%). The *K_i_* values could be determined for two inhibitors, compounds **10** (*K_i_* = 16.4 ± 8.1 μM) and **13** (*K_i_* = 15.1 ± 7.6 μM), which showed a mixed-type mechanism of inhibition. Overall, the identified inhibitors fulfill drug-like criteria and are structurally novel compared with known h15-LOX-2 inhibitors.

## Introduction

Lipoxygenases (LOXs) are nonheme iron enzymes that catalyze the regio- and stereospecific dioxygenation of polyunsaturated fatty acids containing at least two isolated *cis* double bonds, such as arachidonic acid (C20:4, *n* – 6, Δ^5,8,11,14^) and linoleic acid (C18:2, *n* – 6, Δ^9,12^), leading to the formation of a chiral hydroperoxide product.^1−4^ Humans have six LOXs (5-lipoxygenase, 12*S*-lipoxygenase, 12*R*-lipoxygenase, 15-lipoxygenase-1, 15-lipoxygenase-2, and epidermal lipoxygenase-3), which are expressed in different tissues and named according to their product specificities.^1,5,6^ The primary products of the LOX pathways are subsequently converted to several pro- or anti-inflammatory bioactive lipid mediators, including leukotrienes, lipoxins, hepoxilins, eoxins, resolvins, protectins, and others.^1,5^ In addition to playing important roles in the regulation of inflammation, LOXs are associated with biological processes such as cell proliferation and differentiation, modification of lipid–protein complexes, and regulation of the intracellular redox state.^1^ Additionally, LOXs have been implicated in the pathogenesis of many human diseases, including cancer, diabetes, and cardiovascular, pulmonary, and neurodegenerative diseases.^7,8^

**Figure 1:**
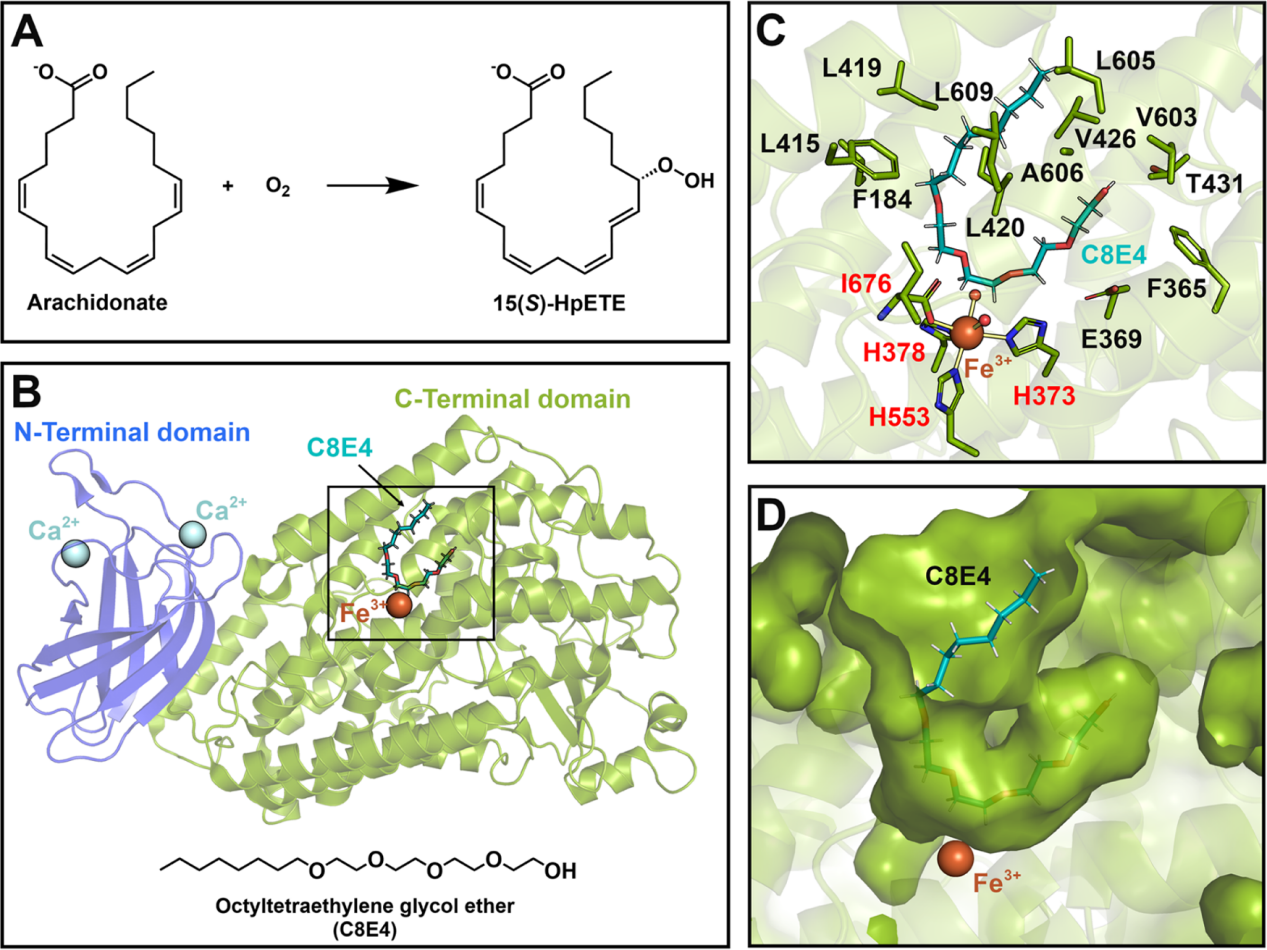
Schematic representations of h15-LOX-2-catalyzed reaction and of h15-LOX-2 overall structure and active site’s cavity. (A) Schematic representation of the main chemical reaction catalyzed by h15-LOX-2, oxygenation of arachidonate to 15*S*-hydroperoxy-(5*Z*,8*Z*,11*Z*,13*E*)-eicosatetraenoate (15(*S*)-HpETE). (B) h15-LOX-2 structure complexed with a substrate mimic inhibitor (octyltetraethylene glycol ether, C8E4) (PDB code: 4NRE; resolution: 2.63 Å).^6^ The amino-terminal β-barrel domain (PLAT domain, membrane-bound domain) and the α-helical carboxy-terminal domain (catalytic domain) are shown in blue and green, respectively. The catalytic iron (Fe^3+^) and two calcium ions (Ca^2+^) that play a role in membrane binding^6^ are shown as brown spheres and as cyan spheres, respectively. The inhibitor C8E4 is represented as sticks. (C) Close-up view of the h15-LOX-2 active site’s cavity. The residue side chains are shown as sticks (carbon, oxygen, and nitrogen atoms in green, red, and blue, respectively). The backbones and hydrogen atoms of the residues have been omitted for clarity. The substrate mimic inhibitor (C8E4)^6^ is represented as sticks (carbon, hydrogen, and oxygen atoms are shown in light blue, white, and red, respectively). Catalytic iron is shown as a brown sphere. Two water molecules that coordinate with Fe^3+^ are shown as red spheres. The residues that coordinate Fe^3+^ (His-373, His-378, His-553, and Ile-676) are labeled in red. Interactions with Fe^3+^ are represented by yellow lines. (D) Surface representation of the h15-LOX-2 active site’s cavity, evidencing its U-shape. The figure was prepared by using PyMOL.

The human 15-lipoxygenase-2 (h15-LOX-2), which is encoded by the *ALOX15B* gene, converts mainly arachidonate to 15*S*-hydroperoxy-(5Z,8Z,11Z,13E)-eicosatetraenoate (15(*S*)-HpETE) (Figure 1A) by attack at C13.^6^ h15-LOX-2 is mainly expressed in macrophages, skin, cornea, lungs, hair roots, and prostate.^9^ In epithelial cells, h15-LOX-2 has been shown to regulate cell senescence.^10^ Recently, the role of h15-LOX-2 in macrophage cholesterol homeostasis has been described in the literature.^11^ Although h15-LOX-2 specific physiological roles have not been completely unveiled, it has been shown that h15-LOX-2 expression levels are higher in human carotid atherosclerotic lesions, in comparison with healthy arteries.^8,12−14^ Additionally, silencing the *ALOX15B* gene in human macrophages has led to a decrease in cellular lipid accumulation and a reduction in proinflammatory cytokine secretion.^15^ It has also been suggested that an increased expression of h15-LOX-2 induced by hypoxia may be associated with chemokine-mediated recruitment of T-cells in atherosclerosis and related diseases.^16^ Therefore, h15-LOX-2 seems to play an important role in the initiation and development of human atherosclerosis.^7,10^ In addition to its roles in atherosclerosis pathogenesis, several studies have suggested that h15-LOX-2 might be implicated in some types of cancers.^7^ h15-LOX-2 expression levels have been shown to be altered in epithelial tumor cells^17−23^ and in tumor-associated macrophages from renal cell carcinoma.^24^ A possible role of h15-LOX-2 (and other LOXs) in ferroptosis, an iron-dependent form of cell death, which is largely associated with lipid peroxidation, has also been proposed in the literature.^25−27^ However, the pharmacological importance of LOXs, including h15-LOX-2, as potential biological targets for ferroptosis-related diseases remains to be better studied.^25−27^

From a structural point of view, a crystal structure of h15-LOX-2 complexed with a substrate mimic inhibitor (octyltetraethylene glycol ether, C8E4, Figure 1B) (PDB code: 4NRE; resolution: 2.63 Å)^6^ reveals that h15-LOX-2 has a cylindrical shape and displays a typical LOX fold, with an amino-terminal β-barrel domain (PLAT domain), which contains two calcium ions that play a role in membrane binding,^6^ and a carboxy-terminal α-helical domain, which contains the substrate binding site and catalytic iron (Figure 1B).^6,9^ The active site of h15-LOX-2 is in a U-shaped cavity formed primarily by hydrophobic amino acid residues (Figure 1C,D), which are expected to interact with the substrate’s nonpolar fatty acid tail in a similar way they interact with C8E4.^6^

**Figure 2:**
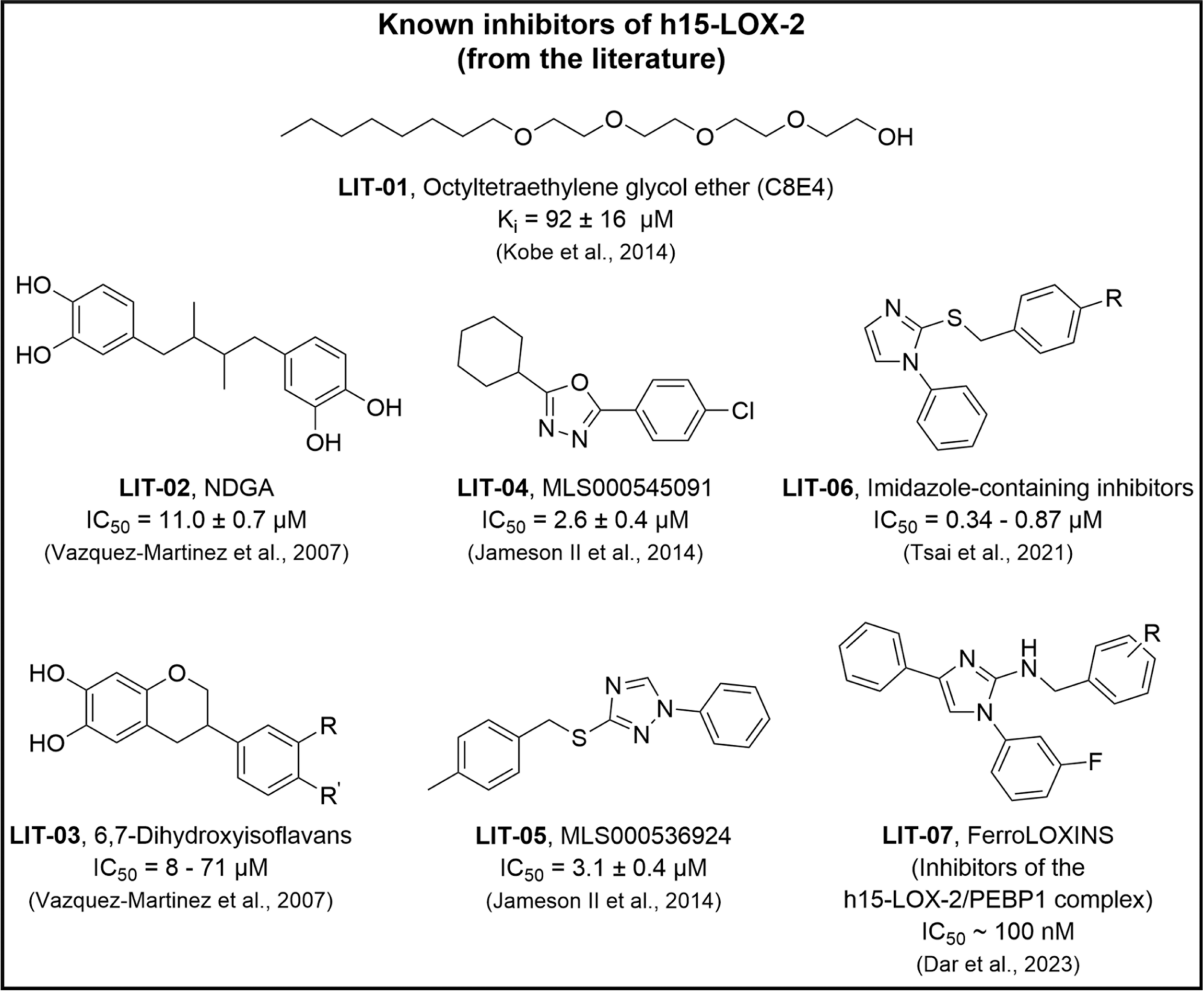
Structures of some h15-LOX-2 inhibitors reported in the literature.^9,28−30^

Considering the complex (and so far poorly understood) biological functions of h15-LOX-2 and its potential as a therapeutic target for human diseases like atherosclerosis and cancer, discovering h15-LOX-2 inhibitors is of great interest for the development of chemical probes and/or of potential drug candidates.^6,9,28^ The elucidation of the h15-LOX-2 three-dimensional (3D) structure by X-ray crystallography^6,9^ has provided the basis for the structure-based design of inhibitors for this target enzyme.^9^ Nevertheless, only a few h15-LOX-2 inhibitors have been reported in the literature so far (Figure 2).^6,9,28−32^ The substrate mimic inhibitor C8E4 (**LIT-01**), mentioned above, is a polyoxyethylene detergent that competitively inhibits h15-LOX-2 with a *K_i_* value of 92 ± 16 μM, occupying the active site’s cavity^6^ (Figure 1B). Despite the similarity with arachidonate, C8E4 lacks the 1,4-*cis*,*cis* pentadiene unit from which a bis-allylic proton is removed in the first step of the h15-LOX-2-catalyzed substrate oxygenation, which makes it an inhibitor rather than a substrate. Additionally, nordihydroguaiaretic acid (NDGA, **LIT-02**) and some flavonoid-based compounds (*e.g.*, **LIT-03**) have been described as moderately potent and nonselective h15-LOX-2 inhibitors, which act by reducing the active site’s catalytic iron.^29^

More recently, a few low-micromolar h15-LOX-2 inhibitors that contain metal-binding groups, such as 1,3,4-oxadiazole (*e.g.*, **LIT-04**),^28^ 1,2,4-triazole (*e.g.*, **LIT-05**),^28^ and imidazole (*e.g.*, **LIT-06**),^9^ have also been reported. These inhibitors show some selectivity against other LOXs (*e.g.*, h15-LOX-1, h5-LOX, and h12-LOX, which have 37, 42, and 37% sequence identities with h15-LOX-2, respectively) and against cyclooxygenases (COX-1 and COX-2).^9,28^**LIT-04** and **LIT-05** have been reported as mixed-type and competitive h15-LOX-2 inhibitors,^28^ respectively, and the structure of h15-LOX-2 complexed with **LIT-05** has been elucidated by X-ray crystallography (PDB code: 7LAF; resolution: 2.44 Å), revealing that it binds to the U-shaped active site’s cavity.^9^ Still, some compounds (*e.g.*, **LIT-07**) have been recently reported to inhibit the catalytic activity of the complex h15-LOX-2/PEBP1 (phosphatidylethanolamine (PE)-binding protein 1).^30^ The association of h15-LOX-2 with PEBP1 is known to play a role in the initiation of ferroptosis-specific peroxidation of polyunsaturated PE (*e.g.*, arachidonic acid-PE), and therefore, inhibitors having the **LIT-07** scaffold have been reported to inhibit the production of PE hydroperoxides, in addition to suppressing ferroptosis in cell culture models.^30^ However, inhibitors of the h15-LOX-2/PEBP1 complex do not act on h15-LOX-2 alone.

Additionally, despite the recent advances in terms of h15-LOX-2 inhibitor development, the few recently described inhibitors of h15-LOX-2 (**LIT-03**, **LIT-04**, and **LIT-05**) and of the h15-LOX-2/PEBP1 complex (**LIT-07**) lack structural diversity since they are all derived from a similar scaffold that consists of a substituted oxadiazole, triazole or imidazole (Figure 2). Moreover, to our knowledge, the few known h15-LOX-2 inhibitors are still in the early stages of development as drug candidates and have not yet reached the market. Therefore, the need for discovering novel h15-LOX-2 inhibitors remains urgent.

Here, we used a virtual screening (VS) protocol, combining ligand-based^33^ and structure-based^33^ approaches, to screen a “curated” version of the ZINC database^34,35^ (prefiltered based on drug-like^36^ properties) for novel, structurally diverse, and potentially drug-like h15-LOX-2 inhibitors. *In vitro* enzyme inhibition assays were carried out for VS experimental validation, resulting in the identification of six novel h15-LOX-2 inhibitors with inhibitory activities in the micromolar range. The identified inhibitors are structurally diverse from the h15-LOX-2 inhibitors reported in the literature so far,^6,9,28−32^ show drug-like properties, and are predicted to interact with the more solvent-accessible arm of the U-shaped active site’s cavity.

## Results and Discussion

**Figure 3:**
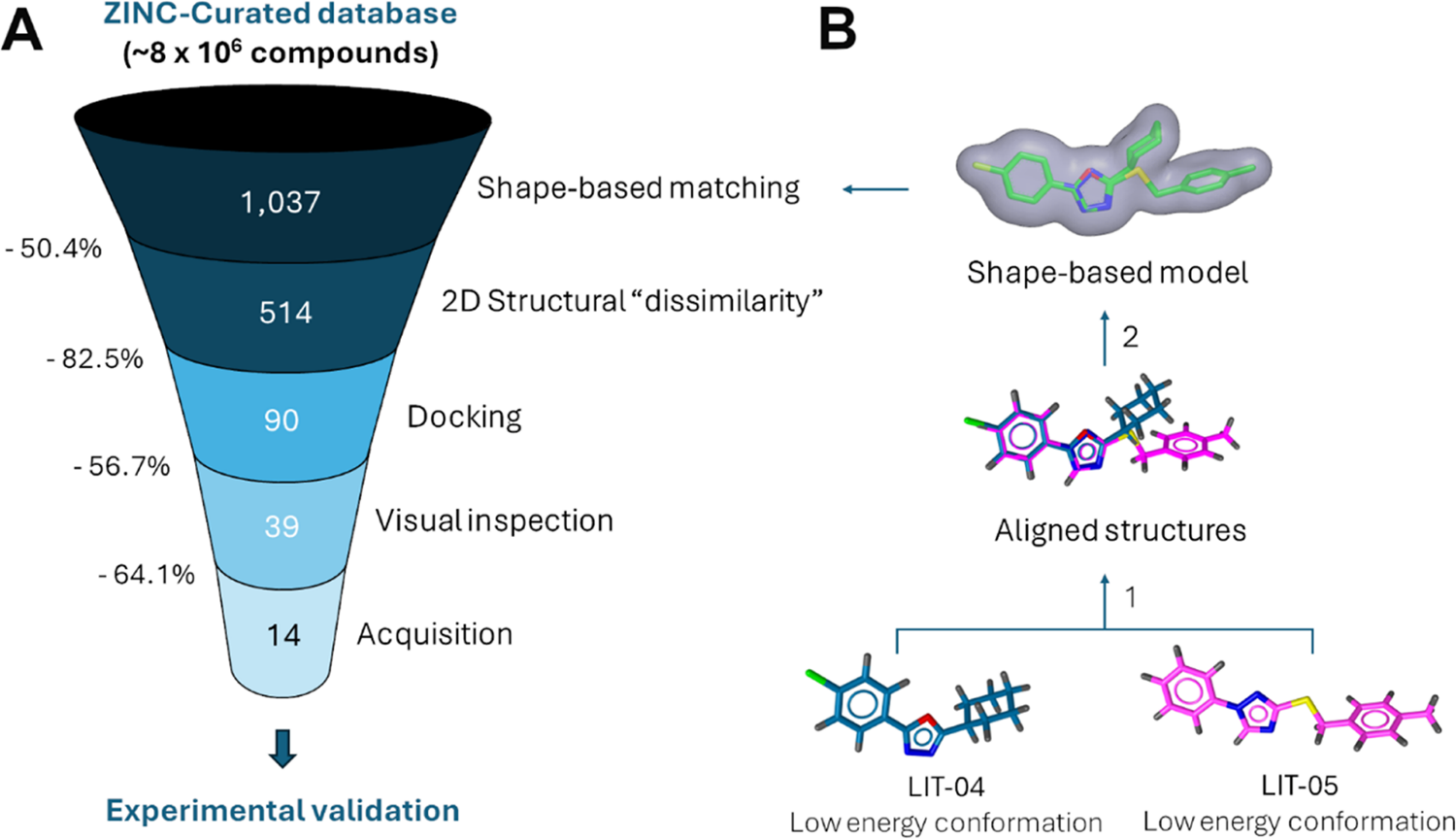
Schematic representation of the virtual screening (VS) protocol used in this study to select novel potential h15-LOX-2 inhibitors from the ZINC-Curated database. (A) Representation of the sequence of selection filters applied to the ZINC-Curated database in each VS step. (B) Schematic representation of the steps to generate the shape-based model that was used as the first filter in the VS protocol: 1. Alignment of low energy conformation structures of two known h15-LOX-2 inhibitors (**LIT-04** and **LIT-05**),^28^ using LigandScout’s alignment algorithm, and 2. Shape-based model generation using ROCS. The molecular shape surface is represented in gray. The figure was prepared by using LigandScout and ROCS.

An overview of the hierarchical VS protocol that was used in this work to search for novel h15-LOX-2 inhibitors is schematically represented in Figure 3A.

### Shape-Based Screening

**Figure 4:**
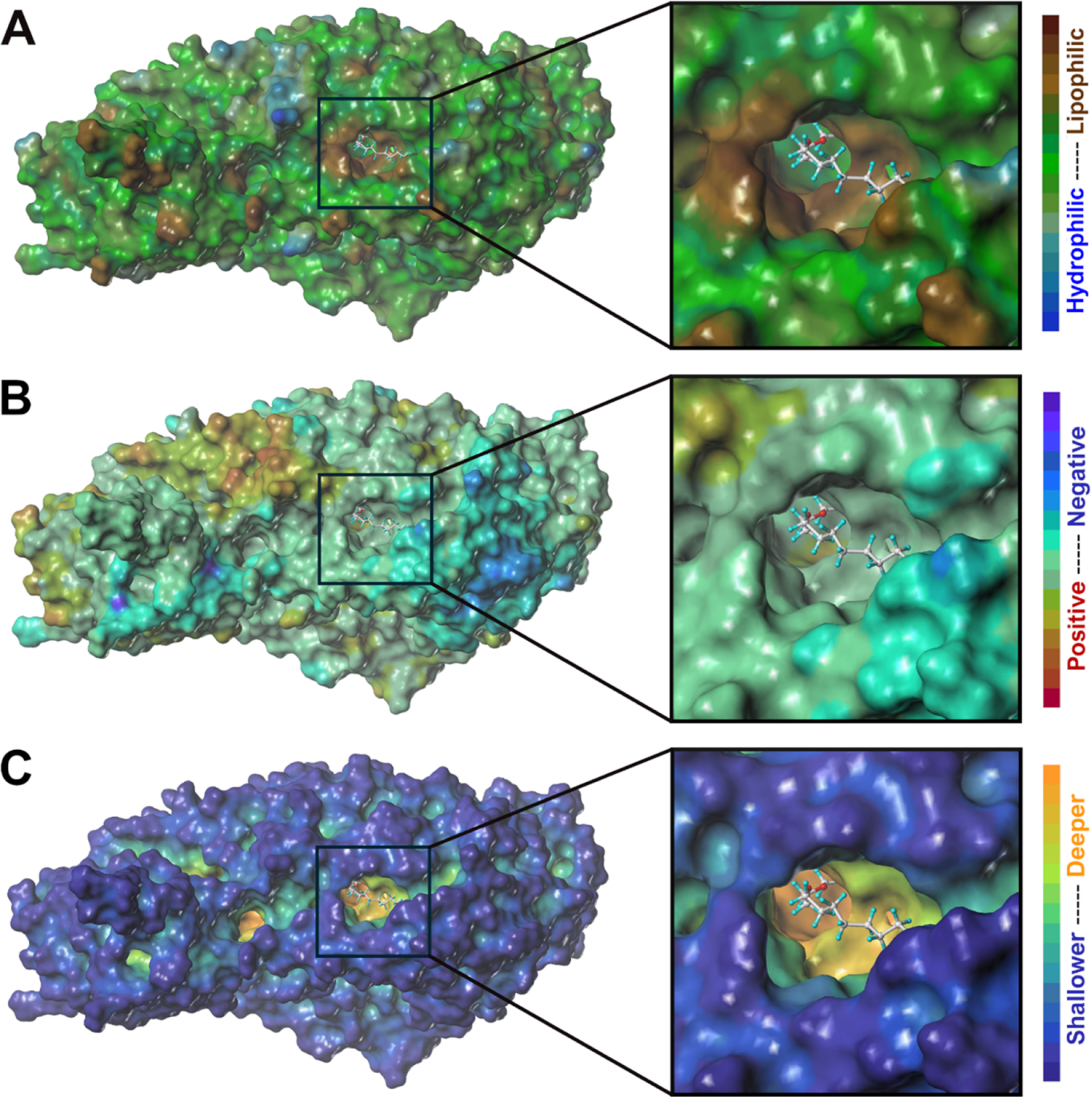
(A) Lipophilic potential, (B) electrostatic potential, and (C) cavity depth maps generated for h15-LOX-2, with a close-up view of the entry to the active site’s cavity. The maps were generated using the MOLCAD^50^ module of SYBYL-X^51^ v.2.1.1, applying the Connolly method^52^ for calculating the protein’s solvent-accessible surface area. The substrate mimic inhibitor C8E4 (**LIT-01**), which was cocrystallized with h15-LOX-2,^6^ is shown as ball-and-sticks (carbon, hydrogen, and oxygen atoms shown in gray, cyan, and red, respectively). The crystal structure of h15-LOX-2 (PDB code: 4NRE; resolution: 2.63 Å)^6^ was used to generate the maps. The figure was prepared by using SYBYL-X.

As discussed in the literature, shape complementarity is one of the most important aspects of protein–ligand recognition,^37−39^ mainly for lipid substrates, which usually bind large, buried, and hydrophobic binding sites inside their target proteins.^40^ This is the case of arachidonate’s U-shaped binding site in h15-LOX-2, which is located in a highly hydrophobic, neutral, and deep cavity, as revealed by lipophilic potential, electrostatic potential, and depth cavity maps generated using SYBYL (Figure 4). Motivated by the recognized importance of shape complementarity for protein–ligand binding, and by the successful applications of shape-based approaches in the search for inhibitors of membrane-bound receptors and of enzymes that bind lipids (see, *e.g.*, references^41−48^) and other types of substrates,^49^ we used a shape-based matching approach as a first filter in our VS protocol for selecting novel h15-LOX-2 inhibitors (Figure 3A). With this purpose, two known and selective h15-LOX-2 inhibitors reported in the literature (referred to as **LIT-04** and **LIT-05**, Figure 2)^28^ were used to build a shape-based model (“query”),^39^ using ROCS^39^ (Figure 3B). The choice of **LIT-04** and **LIT-05** as reference structures to build the shape-based model was motivated by the knowledge about their mechanisms of inhibition. **LIT-04** has been described as a mixed-type inhibitor, with preferential binding to free h15-LOX-2, and **LIT-05** has been described as a competitive inhibitor.^28^ Additionally, a crystal structure of h15-LOX-2 complexed with **LIT-05** is available (PDB code: 7LAF; resolution: 2.44 Å),^9^ confirming that this inhibitor binds to h15-LOX-2 active site’s cavity, and docking calculations reported in the literature have suggested that **LIT-04** also occupies the 15-LOX-2 active site’s cavity.^28^ To build our shape-based model, low energy conformations of **LIT-04** and **LIT-05** were first generated and the structures of these two inhibitors were subsequently aligned to each other (Figure 3B). To validate our shape-based model, the structure of **LIT-05**, in the same conformation as observed in the crystallographic complex with h15-LOX-2 (PDB code: 7LAF; resolution: 2.44 Å),^9^ was applied to the model. As shown in Figure S1, the crystallographic conformation of **LIT-05** fitted the model with a Shape Tanimoto score of 0.508.

A “curated” version of the ZINC database (referred to hereafter as “ZINC-Curated”), prefiltered for potentially drug-like compounds as described in the Experimental Section, was screened for compounds that have a high shape similarity to our proposed shape-based model. Compounds from ZINC-Curated were ranked by ROCS^39^ according to their Shape Tanimoto^39^ score values. The 1,037 compounds with the highest Shape Tanimoto values were selected for further analyses, which represents a reduction of ∼99.9% in the number of compounds from the ZINC-Curated database (Figure 3A). The Shape Tanimoto values for the selected compounds range from 0.516 to 0.565.

### 2D Structural “Dissimilarity”^53^ Filter

A preliminary visual inspection analysis of the structures of the 1,037 compounds selected from the ZINC-Curated database by the proposed shape-based model revealed that they are overall structurally diverse, but small groups of compounds with very similar scaffolds among each other were also identified. Considering that we aim to select inhibitors with as diverse structures as possible, in the second step of our VS we applied a two-dimensional (2D) structural “dissimilarity” filter (Figure 3A) to reduce the number of compounds, keeping the structural diversity at the same time. Proceeding as described in the Experimental Section, structurally similar compounds were first grouped into clusters based on molecular fingerprints.^53^ Using a “dissimilarity” index of 0.15 (*i.e*., a maximum of 85% of structural similarity) as a cutoff value, 514 clusters were generated, and only one representative compound of each cluster was randomly selected for the next VS steps. The 514 compounds selected (one of each cluster) represent 50.6% of the 1,037 compounds that passed the shape-based matching filter, which means that a reduction of 50.4% was achieved with the 2D structural “dissimilarity” filter (Figure 3A).

### Docking and Visual Inspection Analyses

Compounds selected by the shape-based matching and by the 2D “dissimilarity” filters were subsequently docked into the h15-LOX-2 active site, using GOLD.^54^ The docking procedure was previously validated by “redocking” the substrate mimic inhibitor C8E4 (**LIT-01**), which was cocrystallized with h15-LOX-2 (PDB code: 4NRE; resolution: 2.63 Å),^6^ into the h15-LOX-2 active site’s cavity. The redocking was done using each of the four scoring functions available in GOLD^54^ (ChemPLP,^55^ ASP,^56^ Chemscore,^54^ and Goldscore^57^). As shown in Table S1, the RMSD value calculated between the Goldscore best-scored docking pose (pose 1) of **LIT-01** and the crystallographic pose of this inhibitor was the lowest (2.07 Å) compared to the other scoring functions. This value is lower than the resolution value of the complex 15-LOX-2/C8E4 crystal structure^6^ (2.63 Å). Considering the average of the two best-scored docking poses, ChemPLP also performed relatively well with an average RMSD of 2.79 Å (Table S1). A visual inspection of the docking poses confirms that the best-scored poses of both Goldscore and ChemPLP performed well in terms of reproducing the crystallographic binding pose of **LIT-01** (Figure S2). To complement the validation of the docking procedures, the known inhibitor **LIT-05**,^28^ which was used to build our shape-based model and whose binding mode to h15-LOX-2 was determined by X-ray crystallography,^9^ was also submitted to docking into h15-LOX-2 active site’s cavity. As shown in Table S2, the RMSD value calculated between the Goldscore best-scored docking pose (pose 1) of **LIT-05** and the crystallographic pose of this inhibitor was the lowest (3.63 Å) compared to the other scoring functions. The RMSD value for the best-ranked pose obtained using the ChemPLP scoring function was the second lowest (4.56 Å, Table S2). A visual inspection of the docking poses suggests that, even though the binding mode of **LIT-05** observed in the crystallographic structure was not reproduced using either ChemPLP or Goldscore, the binding modes predicted using these scoring functions are reasonable, suggesting a possible cation-π interaction between the electron-rich π system of the methyl-benzene moiety and the catalytic iron ion (Figure S3). Therefore, considering the relatively good performance of Goldscore and ChemPLP in the redocking of **LIT-01** and **LIT-05**, these two scoring functions were chosen to perform the docking calculations in our VS protocol.

**Figure 5:**
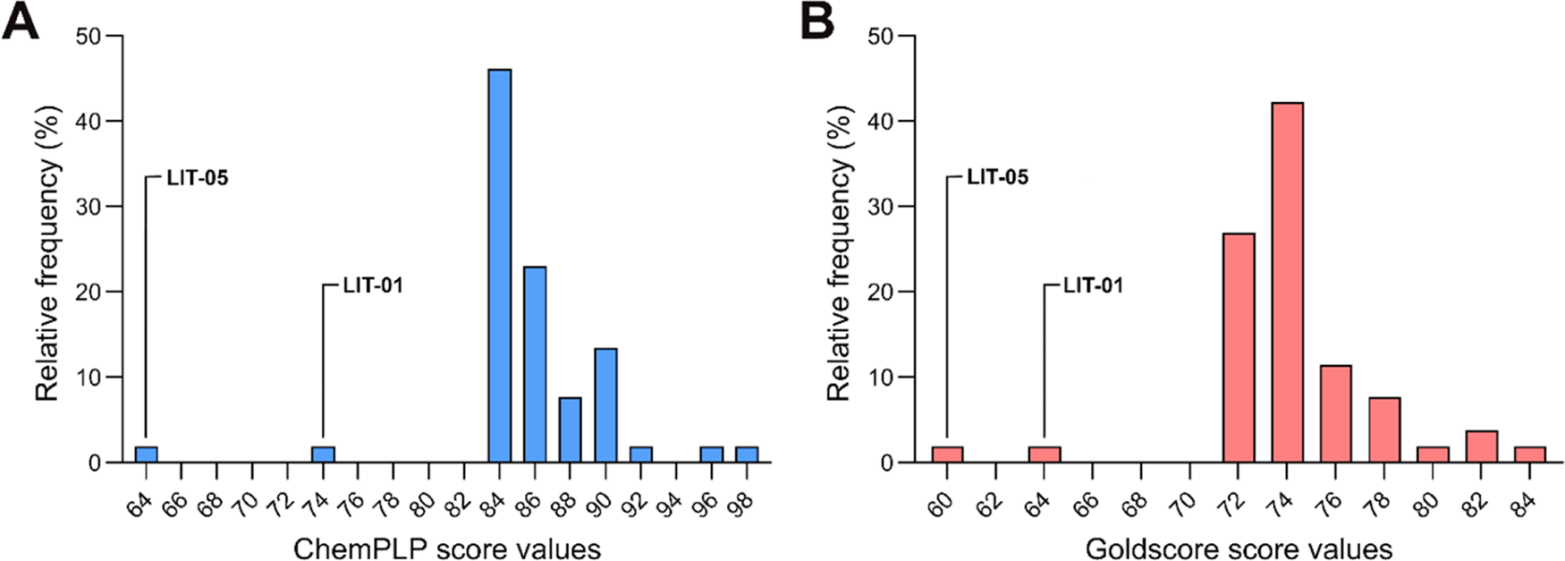
Score frequency distributions of the best-ranked docking poses for the compounds selected from the ZINC-Curated database after applying the docking filter. (A) ChemPLP score values distribution. (B) Goldscore score values distribution. The figure was prepared using GraphPad Prism. The ChemPLP and Goldscore score values of two h15-LOX-2 known inhibitors (**LIT-01**^6^ and **LIT-05**(28)) are represented for comparison.

Among the 514 compounds submitted to docking into the h15-LOX-2 active site’s cavity, the 50 compounds with the highest ChemPLP score values (ranging from ∼83 to ∼98) and the 50 compounds with the highest Goldscore score values (ranging from ∼72 to ∼84) were selected for subsequent analysis. As 10 compounds were among the 50 best-scored using both ChemPLP and Goldscore, a total of 90 compounds were selected for the next step of our VS protocol, which represents a reduction of ∼82.5% in the number of compounds that passed the 2D structural “dissimilarity” filter step (Figure 3A). The ChemPLP and Goldscore score values distributions of the compounds selected by the docking filter are shown in Figure 5. Remarkably, the cutoff score value for ChemPLP (83.23) is ∼4 units above the third percentile of the ChemPLP scores computed from all docked compounds (78.99; Table S3). The same observation is valid for the compounds selected using the Goldscore scoring function (cutoff score value of 71.73 and third percentile of 67.62) (Table S3). Compounds that did not pass the docking filter had score values ranging from ∼54 to ∼83 (ChemPLP) and from ∼6 to ∼72 (Goldscore).

Notably, the cutoff score values of the selected compounds are higher than the score values for the best-scored poses of the known inhibitors **LIT-01**(6) (74.13 and 63.92 with ChemPLP and Goldscore, respectively) and **LIT-05** (64.36 and 59.90 with ChemPLP and Goldscore, respectively), which were used for the docking validation procedure, as discussed above. This suggests that even considering that docking scoring functions have intrinsic limitations,^58^ the cutoff score values used in our docking filter seem suitable for selecting compounds with a high probability of being true inhibitors.

In the last step of the proposed VS protocol, the best-scored pose of each compound selected by the docking filter was carefully analyzed by visual inspection inside the h15-LOX-2 active site’s cavity, using the LigandScout^59^ program for recognizing protein–ligand interactions. In this step, aiming to select the compounds that best fit into the h15-LOX-2 active site’s cavity, the following criteria were applied: (i) there should be hydrophobic interactions with at least four active site residues; (ii) there should be at least one hydrogen-bond and/or an ionic interaction and/or a π-cation and/or a halogen bond interaction with at least one active site’s residue and/or with the catalytic iron and/or a water molecule; (iii) the best-scored docking poses should be “reproducible”, *i.e*., there should be at least four other poses, among the 10 docking poses, with similar conformations and orientations inside the active site’s cavity; and (iv) there should be a shape complementarity between the compound and the enzyme’s active site.

The structures of the selected compounds and some of their physicochemical properties are shown in Table S4. Among the 39 compounds selected from the ZINC-Curated database by our VS protocol, 14 compounds (∼35.9%) were acquired and tested through *in vitro* enzymatic assays for VS experimental validation (Figure 3A). Among the 39 selected compounds, 23 were selected with only Goldscore, 9 were selected with only ChemPLP, and 7 were selected with both scoring functions. Notably, the average score values of compounds that matched the visual inspection criteria (88.75 and 75.29 with ChemPLP and Goldscore, respectively; Table S5) are significantly higher than the average score values of compounds that did not match the criteria (85.15 and 73.76 with ChemPLP and Goldscore, respectively; Table S5), according to the unpaired *t* test (*p* < 0.05).

### Enzymatic Inhibition Assays for VS Experimental Validation and Discussion on the VS Approach

To evaluate the inhibitory activities of the 14 acquired compounds (**01**-**14**, Table 1), *in vitro* inhibition assays with purified h15-LOX-2 were performed, using a UV-based method for detecting 15(*S*)-HpETE, the conjugate diene-containing enzyme-catalyzed reaction product, at λ = 234 nm. h15-LOX-2 was expressed in *Escherichia coli* BL21 DE3 pLysS cells and purified as described in the Experimental Section. A representative result of the SDS-PAGE analysis of h15-LOX-2 purified samples is shown in Figure S5. The values of the kinetic parameters *K*_M_, *V*_max_, and *k*_cat_, which were determined using arachidonic acid as a substrate, showed good agreement with the literature^6^ (Figure S6 and Table S6). Dimethyl sulfoxide (DMSO) was used as a solvent for the tested compounds, with a final concentration of 1.0% (v/v) in all assays. This DMSO concentration did not significantly affect the h15-LOX-2 activity (Figure S7). All assays were performed in the presence of the nonionic detergent Triton X-100 (at 0.01%, v/v) to avoid compound aggregate formation, which is recognized as an important source of false-positive results in enzyme inhibition screening assays, as discussed in the literature.^60−64^ At a concentration of 0.01% (v/v), Triton X-100 was relatively well tolerated by h15-LOX-2 (Figure S8). Compound **LIT-02** (NDGA, Figure 2), a known inhibitor of h15-LOX-2,^29^ was used as a positive control of the enzymatic assays (concentration–response curve in Figure S9). The experimentally determined IC_50_ value of 3.0 ± 1.1 μM for this inhibitor shows a reasonable agreement with the IC_50_ value reported in the literature (11.0 ± 0.7 μM).^29^

**Table 1:**
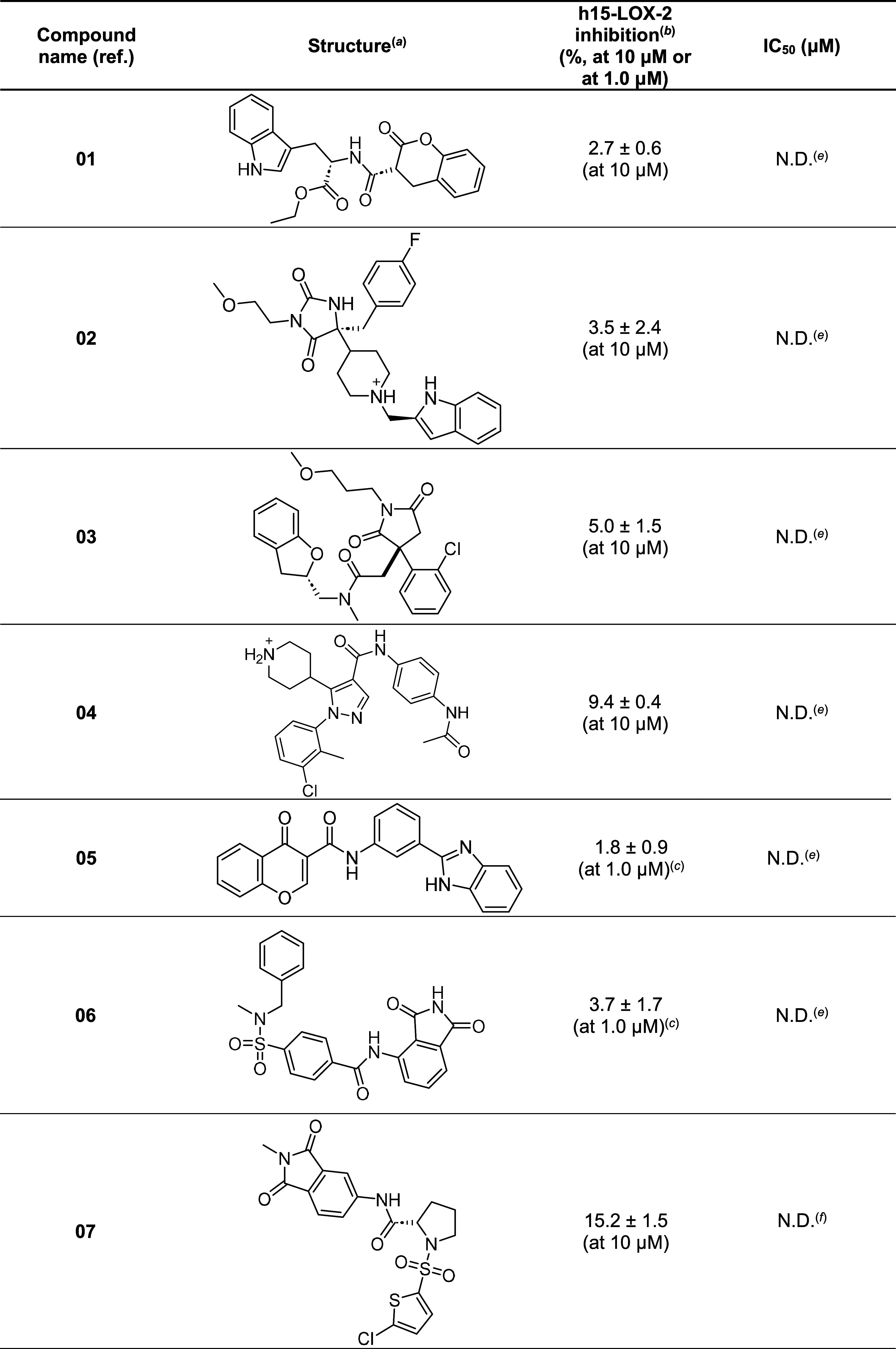

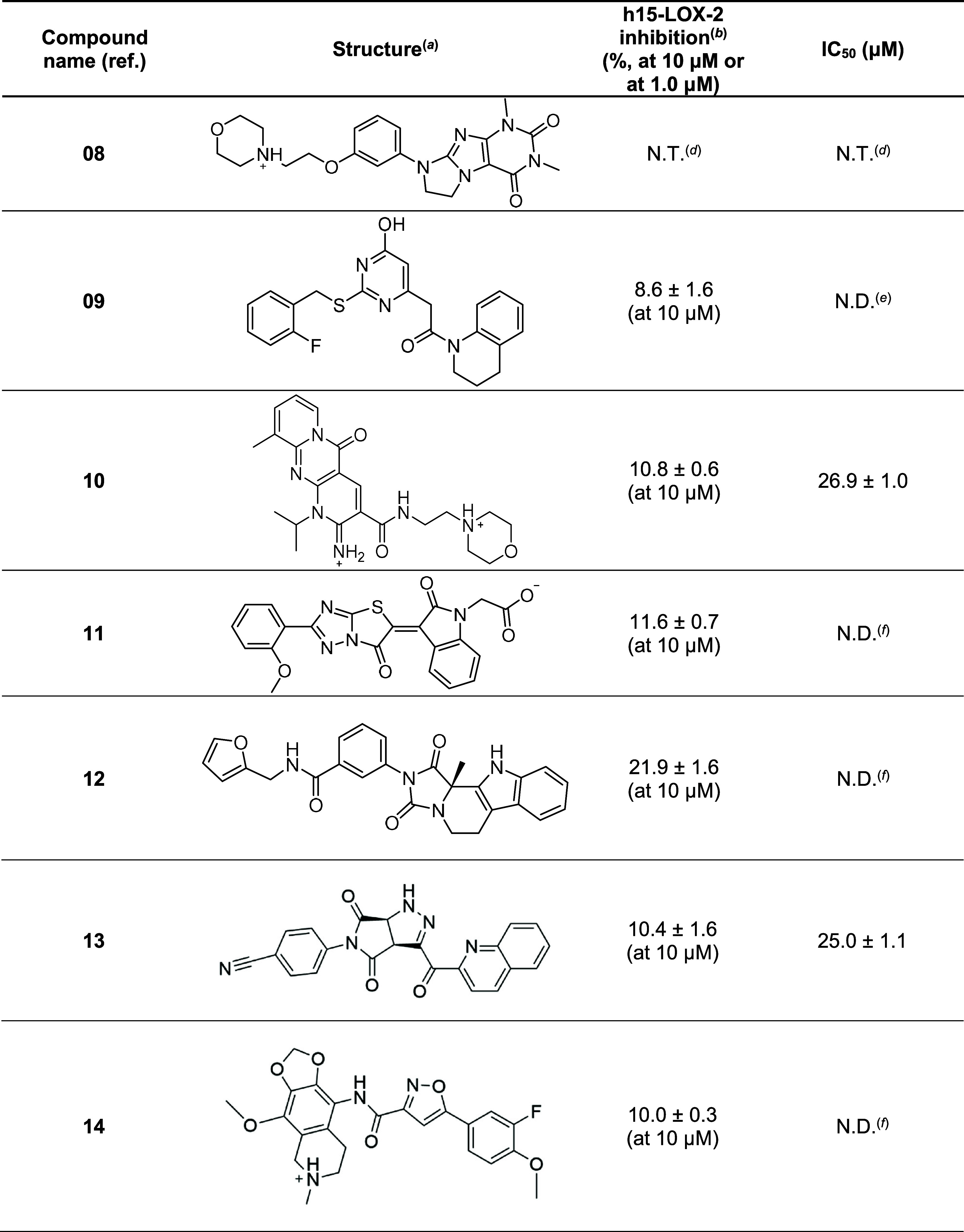
Structures and Inhibitory Activities (Percentages of Inhibition at 10 or 1 μM) of the Compounds Selected as Potential h15-LOX-2 Inhibitors Using the VS Protocol Proposed in this Study

a Structures are represented considering the most abundant protonation states for ionizable groups at pH = 7.4, according to MoKa and/or FixpKa p*K*_a_ predictions.

b Assay conditions: Tris buffer (25 mM, pH = 8.0), NaCl (250 mM), h15-LOX-2 (420 nM), Triton X-100 (0.014% v/v), arachidonic acid (25 μM), DMSO (1.0% v/v). Data are expressed as average ± SEM. All assays were performed at least in triplicates.

c These compounds were tested at 1 μM due to their limited solubilities in the assay buffer (<10 μM).

d Not tested due to the low solubility in DMSO (<500 μM).

e The IC_50_ values of these compounds were not determined because their percentages of inhibition (at 10 μM or at 1 μM) were below the cutoff of 10%.

f The IC_50_ values of these compounds could not be accurately determined due to their low solubilities in the assay buffer (overall <200 μM; see concentration–response curves in Figure 6 and data in Table S7). Compounds **07** and **11** showed more than 50% of inhibition at 25.1 μM and at 63.1 μM (66.4 ± 3.7 and 64.7 ± 0.5%), respectively.

Initially, all 14 compounds were tentatively tested at 10 μM (or at 1 μM, depending on the solubility in the assay buffer; Table 1). Compound **08** could not be tested, because it was not soluble in DMSO, which was used as a solvent for preparing stock solutions of all tested compounds, as stated above. Six compounds (**07**, **10**, **11**, **12**, **13**, and **14**) inhibited h15-LOX-2 activity in at least 10% at 10 μM, and concentration–response curves were obtained for five of them (**07**, **10**, **11**, **12**, and **13**; Figure 6). Compounds **10** and **13** showed IC_50_ values of 26.9 ± 1.0 and 25.0 ± 1.1 μM, respectively (Table 1). Due to the limited solubilities of **07** (between 50 and 79 μM), **11** (between 100 and 125 μM), and **12** (between 13 and 20 μM) in the assay buffer (Table S7), the IC_50_ values of these compounds could not be obtained from their concentration–response curves (Figure 6). Nonetheless, it was observed that **07** and **11** showed more than 50% inhibition at 25.1 μM and at 63.1 μM (66.4 ± 3.7 and 64.7 ± 0.5%), respectively (Table 1). Considering that **14** showed a low inhibitory activity at 10 μM (close to the cutoff of 10%) and a very low solubility in the assay buffer (between 32 and 50 μM), a concentration–response curve was not obtained for this compound. It was checked, using FILTER^65−67^ toolkits, that none of the active compounds had a “pan assay interference compound” (PAINS)^68,69^ substructure.

**Figure 6:**
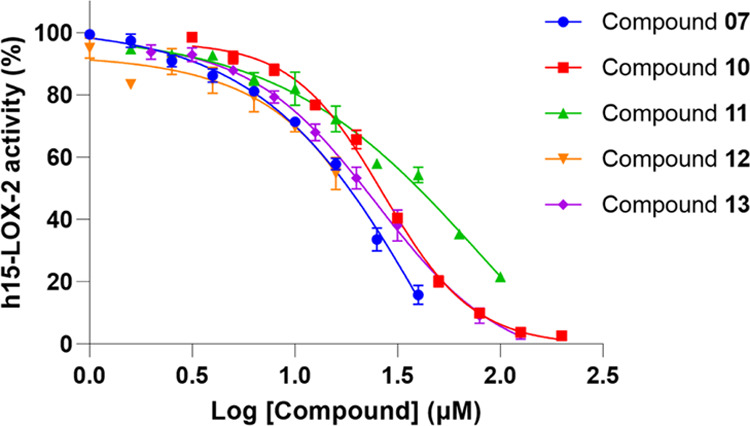
Concentration–response curves for compounds **07, 10, 11, 12, and 13**. All assays were carried out in a total volume of 300 μL, containing Tris buffer (25 mM, pH = 8.0), NaCl (250 mM), h15-LOX-2 (420 nM), Triton X-100 (0.014% v/v), arachidonic acid (25 μM), and each tested compound (1.0 μM to up to 199.1 μM, depending on the compound solubility in the assay buffer). Data represent the average ± SEM of experiments performed at least in triplicates. For compounds **10** and **13**, data represent the average of two independent assays, one performed in triplicate and one performed in duplicates. Complete concentration–response curves could not be obtained for compounds **07**, **11**, and **12**, due to their low solubilities in the assay buffer (<200 μM). The determined Hill slope values for each concentration–response curve were as follows: compound **07** (−1.0 ± 0.3), compound **10** (−2.0 ± 0.1), compound **11** (−0.9 ± 0.3), compound **12** (not accurately determined), and compound **13** (−1.4 ± 0.2). The figure was prepared using GraphPad Prism.

The low solubilities of some inhibitors selected by our VS are expected, even though we used solubility filters based on *in silico* predictions^70^ in the preparation of the ZINC-Curated database (see Experimental Section). First, as discussed in the literature,^40^ the lipophilic nature of the substrate binding pockets in fatty acid-binding proteins, such as the h15-LOX-2 active site’s cavity (see Figure 4A), poses a considerable challenge in terms of design of soluble and “drug-like” inhibitors. Second, despite the advances in the field, accurate *in silico* prediction of solubility remains a big challenge due to the complexity of the chemical and physicochemical factors that influence solubility,^70−73^ including the nature, temperature, pH, and ionic strength of the solvent, solid state of the solute (amorphous or crystal), solute polymorphisms, intermolecular interactions between the solute and the solvent, ionization state(s) of the solute, among others. Despite the solubility issues, compounds **07**, **11**, **12**, and **14**, as well as the other compounds selected by our VS and tested through enzymatic assays, passed the “drug-like” filter criteria that were used for building our ZINC-Curated database. Additionally, the identified inhibitors (including compound **14**) have structural and physicochemical properties that are overall inside the suitable physicochemical space for a good chance of oral bioavailability, according to Lipinki’s Rule of Five^74,75^ and other empirical rules such as implemented in SwissADME^76^ (Figure S4). However, it is important to stress that physicochemical descriptors used to define empirical rules do not substitute experimental data and, therefore, should not be used to make definitive conclusions about bioavailabilities and drug-likeness.^36,77^*In vitro* and *in vivo* studies are still needed to characterize the bioavailabilities of the identified inhibitors, and eventual structural optimization steps might be necessary to improve properties that influence their bioavailabilities such as solubility in aqueous media.

**Figure 7:**
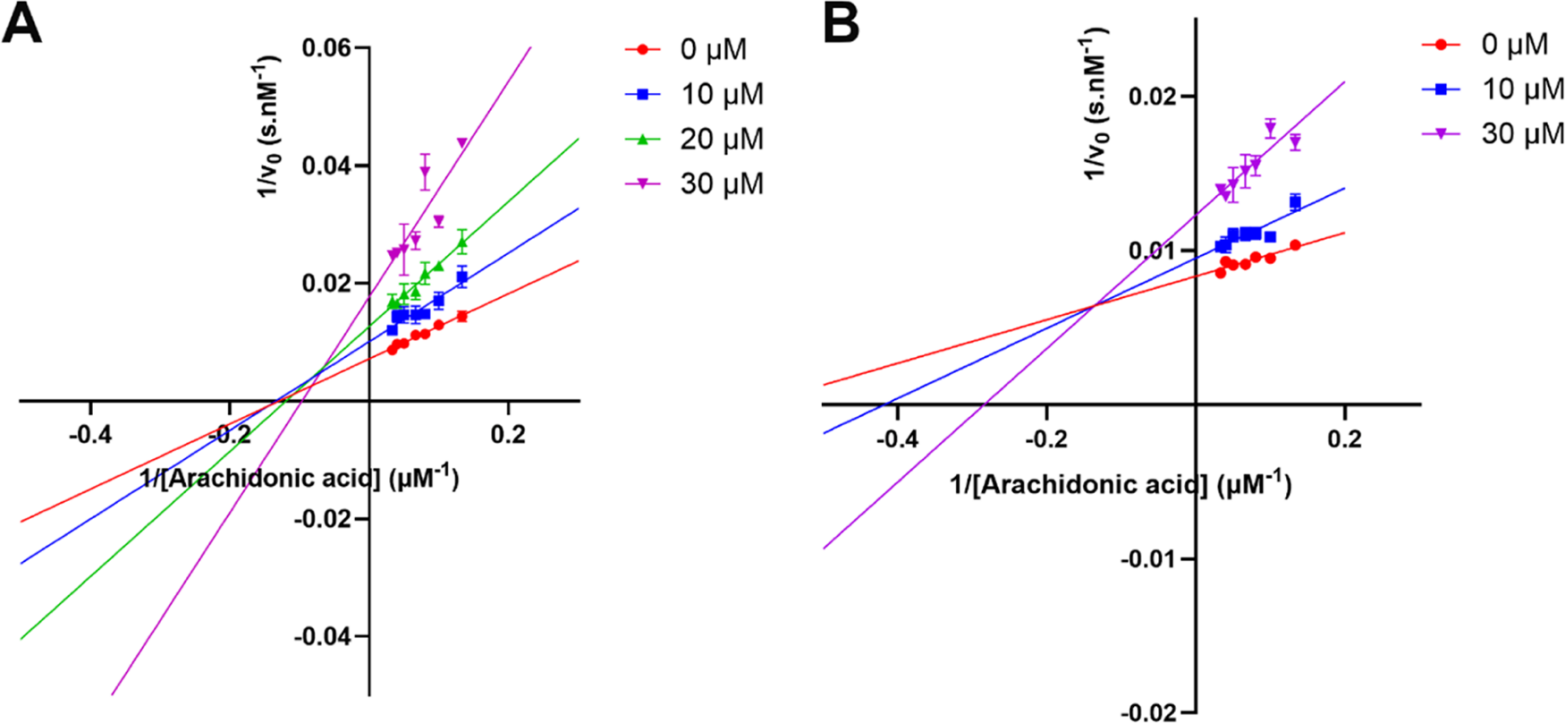
Lineweaver–Burk plots for (A) compound **10** and (B) compound **13**. All assays were carried out in a total volume of 300 μL, containing Tris buffer (25 mM, pH = 8.0), NaCl (250 mM), h15-LOX-2 (420 nM), Triton X-100 (0.014% v/v), arachidonic acid (7.5 μM - 30 μM), and the tested compound (10 μM, 20 μM, and/or 30 μM). Data represent the average ± SEM of experiments performed at least in triplicates. The figure was prepared using GraphPad Prism.

**Figure 8:**
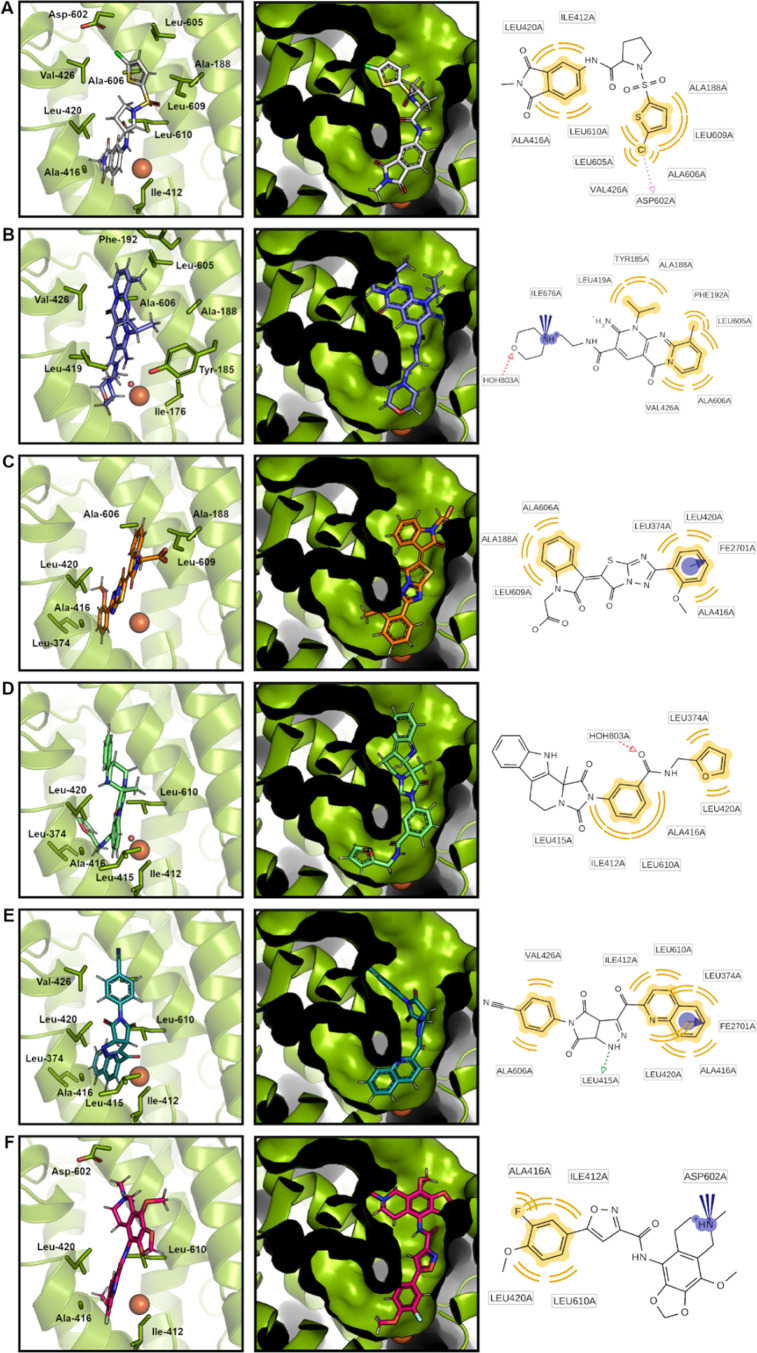
Schematic representations of the predicted binding modes (best-scored docking solutions) of the h15-LOX-2 inhibitors selected by the VS protocol proposed in this study: (A) Compound **07**; (B) compound **10**; (C) compound **11**; (D) compound **12**; (E) compound **13**; and (F) compound **14**. The crystal structure of h15-LOX-2 (PDB code: 4NRE; resolution: 2.63 Å) was used for all docking calculations. *Left panels*: Schematic representations of the best-scored docking solutions for each inhibitor inside the h15-LOX-2 active site’s cavity. For clarity, only the active site residues that interact with each inhibitor are highlighted (shown as sticks). Catalytic iron is shown as a brown sphere. A water molecule that coordinates iron and makes a hydrogen-bond interaction with compounds **10** and **12** is shown as a red sphere in panels (B, D). *Central panels*: Schematic representations of the best-scored docking solutions for each compound, with h15-LOX-2 active site’s cavity represented as a surface to highlight its U-shaped format. The inhibitors occupy the more solvent-exposed arm of the cavity, which corresponds to the cavity’s right arm in the protein’s orientation captured to prepare this figure. *Right panels*: 2D Schematic representations of the protein–ligand interactions identified using the LigandScout^59^ program. Yellow: hydrophobic interactions; blue circle/arrow: pi-cation interactions; blue cones: ionic interactions; red arrows: hydrogen-bond acceptor interactions; pink arrow: halogen bond interaction. The figure was prepared using PyMOL.

Lineweaver–Burk plots revealed a mixed-type inhibition for compounds **10** and **13** (Figures 7 and S10), with *K_i_* values of 16.4 ± 8.1 and 15.1 ± 7.6 μM, respectively, and α values of 1.5 ± 1.1 and 4.3 ± 2.8, respectively. This suggests that **10** and **13** are expected to bind to both free h15-LOX-2 and the h15-LOX-2-substrate complex (see Scheme S1 for a simplified schematic representation of a well-accepted mixed-type inhibition model). The α value of 4.3 ± 2.8 for compound **13** (higher than 1.0 even considering the experimental error) indicates that this compound has a greater affinity (∼4.3 times higher) to free h15-LOX-2 than to the h15-LOX-2-substrate complex. The α value of 1.5 ± 1.1 for compound **10** suggests that it probably has a higher affinity to free h15-LOX-2, like **13**, but the possibility of preferential binding of **10** to the h15-LOX-2-substrate complex cannot be completely discarded considering the experimental error. As discussed in the literature,^78−80^ there are several molecular mechanisms of protein-inhibitor binding that would be compatible with a mixed-type inhibition model, which is also treated as a special case of “noncompetitive” inhibition by some authors.^78−80^ Regardless of the molecular mechanism, old and recent studies and reviews from the literature suggest that mixed-type inhibitors would occupy, at least partially, the enzyme’s substrate binding site.^78−80^ Therefore, considering that we aimed to select compounds that bind the h15-LOX-2 active site’s cavity, which accommodates the fatty acid substrate, the experimentally evidenced mixed-type inhibition of **10** and **13** are compatible with the predicted binding modes of these two inhibitors to free h15-LOX-2 (Figure 8B,E). The possible binding modes of **10** and of **13** to the h15-LOX-2-substrate complex, however, are less obvious and cannot be predicted based only on our docking models presented in Figure 8. Compounds **10** and **13** probably bind to the h15-LOX-2-substrate complex at an allosteric site in h15-LOX-2. One might consider the possibility of binding to an allosteric site described in the literature,^81^ located between h15-LOX-2’s C- and N-terminal domains (far from the active site’s cavity, see Figure S11), which would allow for concomitant binding of the substrate. To check this possibility, compounds **10** and **13** were docked into this h15-LOX-2’s allosteric binding site. The docking results reveal that **10** and **13** fit the allosteric binding site, showing shape complementarity with this binding site and interacting mainly with residues from the C-terminal domain (Figure S11). Notably, the Goldscore score values of the docking poses of **10** and **13** into the active site’s cavity are higher than the Goldscore score values of the docking poses into the allosteric binding site (78.32 and 74.17 for compound **10** into the active site’s cavity and into the allosteric binding site, respectively, and 74.07 and 68.35 for compound **13** into the active site’s cavity and into the allosteric binding site, respectively). This is in agreement with the experimental data on the mechanism of inhibition because **10** and **13** probably have a higher affinity to free h15-LOX-2 than to the h15-LOX-2-substrate complex, as discussed above. Nevertheless, the binding of **10** and **13** to the allosteric binding site is to be experimentally confirmed.

According to docking predictions, all six compounds that showed inhibitory activities higher than 10% at 10 μM (compounds **07**, **10**, **11**, **12**, **13**, and **14**) interact with the h15-LOX-2 active site’s cavity residues mainly *via* hydrophobic interactions (Figure 8). This is expected, considering that this cavity is highly lipophilic (as shown in Figure 4A) and formed by the side chains of several hydrophobic amino acids, such as Ala-188, Leu-374, Ile-412, Leu-415, Leu-420, Ala-416, Val-426, Leu-605, Ala-606, Leu-609, and Leu-610 (Figure 1C), which accommodate the lipid substrate arachidonic acid. The identified inhibitors are predicted to occupy the more solvent-exposed arm of the U-shaped active site’s cavity (Figure 8, central panels) and possibly block the substrate access to the entrance of this cavity. Notably, π-cation interactions are predicted to occur between the h15-LOX-2 catalytic iron and the aromatic systems of **11** and of **13** (Figure 8C,E). An ionic interaction is expected between the positively charged nitrogen of the morpholine ring in compound **10** and the carboxylate group of C-terminal Ile-676, and a hydrogen bond could be established between the oxygen of the ring and an iron coordinating water molecule (Figure 8B). Shape complementarity between the identified inhibitors and their binding sites inside the active site’s cavity was clearly observed in the visual inspection analysis, which reinforces the effectiveness of our shape-based approach used as the first filter in the proposed VS.

Considering that selectivity against other LOXs is an important aspect of h15-LOX-2 inhibitor design, we docked the six identified h15-LOX-2 inhibitors into the active sites’ cavities of 15-LOX-1 from rabbit reticulocytes (∼81% sequence identity with the human 15-LOX-1), h5-LOX, and h12-LOX, which show 37, 42, and 37% sequence identities with h15-LOX-2, respectively. The ChemPLP and Goldscore scores of the inhibitors docked into the active sites of these three LOXs were overall lower than their scores into h15-LOX-2 active site’s cavity (Table S8), which suggests that the identified inhibitors would have higher affinities to h15-LOX-2 compared to the other LOXs. These results, however, should be interpreted with caution and experimentally confirmed, once docking scoring functions are intrinsically limited and do not consider all relevant thermodynamic aspects for an accurate quantitative prediction of protein–ligand binding affinities.^58^

Finally, one should notice that the identified inhibitors have diverse structures from each other and from the h15-LOX-2 inhibitors reported in the literature (Figure 2),^9,28,29^ which maximizes the number of scaffolds that can be used as starting points for a hit-to-lead optimization project.^82^ To get some insights into structural modifications that might result in an increase in the inhibitory activities of the identified inhibitors, we used SZMAP and GamePlan calculations^83^ to identify positions in the ligand that might be modified or where the attachment of polar and/or apolar substituents would hypothetically result in a gain of affinity to h15-LOX-2 (see Figure S12). Regardless of the inhibitory potencies of the identified inhibitors, the achieved structural diversity is highly desirable in a VS campaign.^82^ This structural diversity is particularly relevant in the search of h15-LOX-2 inhibitors, considering that h15-LOX-2 is a very underexploited drug target and that there is a small number of h15-LOX-2 inhibitors described in the literature so far, with most deriving from similar scaffolds.^9,28,29^ The success in the selection of structurally diverse inhibitors in our VS is related to the choice of filter that we used. As presented, a shape-based approach was used as the first selection filter. It is known that a major advantage of a shape-based approach is that it allows for selecting compounds having different scaffolds, despite sharing a similar shape.^39,49,84,85^ We additionally improved the structural diversity of the data set by using a 2D structural “dissimilarity” approach to group similar compounds into clusters, aiming to eliminate compounds that had more than 85% of similarity among each other.

## Conclusions

In summary, in the VS presented here, we successfully combined ligand-based approaches^33^ (shape-based matching and a 2D “dissimilarity” filter) and a structure-based approach^33^ (docking followed by careful visual inspection) to screen a “curated” version of the ZINC database, prefiltered for drug-like compounds (here called “ZINC-Curated”), for novel h15-LOX-2 inhibitors. Two inhibitors, **10** and **13**, with *K_i_* values of 16.4 ± 8.1 and 15.1 ± 7.6 μM, respectively, were identified, revealing a mixed-type mechanism of inhibition. Four additional compounds (**07**, **11**, **12**, and **14**) were found to inhibit h15-LOX-2 in the micromolar range, but the IC_50_ and *K_i_* values of these compounds could not be accurately calculated due to their low solubilities in the enzymatic assays buffer. The inhibitors identified in this study are structurally diverse from each other and from the h15-LOX-2 inhibitors reported in the literature,^9,28,29^ are expected to have good oral bioavailabilities based on empirical rules,^74,75,86^ and do not have PAINS^68,69^ substructures. Binding modes predicted by docking reveal that, not surprisingly, all identified inhibitors interact with the h15-LOX-2 active site’s cavity residues mainly by hydrophobic interactions, and they possibly occupy the more solvent-exposed arm of the U-shaped enzyme’s active site cavity, blocking the entrance of arachidonic acid to this cavity. Biophysical studies, using experimental techniques like X-ray crystallography, surface plasmon resonance spectroscopy, and/or isothermal titration calorimetry, should be considered for an in-depth characterization of the binding modes of the identified inhibitors. Despite this being outside the scope of our study, the inhibitory activities of the identified inhibitors make them useful as starting points for hit-to-lead optimization projects aiming at improving bind affinities and, eventually, selectivities and pharmacokinetic properties. From a biological perspective, *in vitro* assays for evaluating the effects of the identified inhibitors in lipid accumulation, h15-LOX-2-derived lipid mediators’ distribution, and foam cell formation in cell-based atherosclerosis models might be considered as a further step. Studies in this direction are underway.

Finally, it is important to stress that, to the best of our knowledge, this study was the first to exploit a shape-based approach to search for novel h15-LOX-2 inhibitors. Considering the importance of shape complementarity for protein–ligand recognition, especially inside the lipophilic and buried binding sites of proteins that bind lipids, such as h15-LOX-2, the choice for a shape-based approach as a first filter in our VS protocol has been crucial for VS success. Given the increasing evidence on the possible role of h15-LOX-2 in atherosclerosis^10−16^ and in other diseases and pathophysiological processes,^9,18,22,24^ including inflammation, some types of cancer and, possibly, ferroptosis, the here reported identification of novel inhibitors of this so-far underexploited target enzyme is of major importance.

## Experimental Section

### Computational Programs

Babel^87,88^ v.2.2.3 (Open Babel Development Team) was used to generate molecular fingerprints; FILTER module of OMEGA^65−67^ v. v.2.5.1.5/v.4.1.2.0 (Openeye Scientific Sofware Inc., Santa Fe, NM) was used to filter the compounds database for drug-like properties and to check the presence of PAINS substructures in the structures of the identified inhibitors; GOLD^54,89^ v.5.2 (*CCDC*, *T*he Cambridge Crystallographic Data Center, Cambridge, U.K.) was used for docking; GraphPad Prism^90^ v.9/v.10 (GraphPad Software, Boston, MA) was used for experimental data analysis; LigandScout^59,91^ v.4.4.7 (Inte:Ligand GmbH, Maria Enzersdorf, Austria) was used for protein–ligand interaction recognition; MoKa^92,93^ v.2.6 (Molecular Discovery Ltd.., Borehamwood, U.K.) was used to attribute protonation states based on *in silico* predictions of p*K*_a_ values; OMEGA^65−67^ v.2.5.1.5 (Openeye Scientific Sofware Inc.., Santa Fe, NM) was used for conformers generation; Open Babel^87,94^ v.2.3.1 (Open Babel Development Team) was used to generate 3D structures of compounds; PyMOL^95^ v.2.5.0 (Schrödinger Inc.., New York, NY) was used for protein’s structures visualization; QUACPAC^96^ v.2.0.0.3 (Openeye Scientific Sofware Inc., Santa Fe, NM) was used to generate tautomers and to attribute protonation states; ROCS^39,97^ v.3.3.0.3/v.3.4.3.0 (Openeye Scientific Sofware Inc.., Santa Fe, NM) was used to perform shape-based screening; SwissADME^76,98^ (University of Lausanne, Lausanne, Switzerland) was used for physicochemical properties and drug-likeness predictions; SYBYL-X^51^ v.2.1.1 (Certara Inc.., PA) was used for protein’s structure preparation for docking procedures and for protein’s solvent-accessible area calculations.

### Protein Structure Preparation for the Virtual Screening Procedures

The h15-LOX-2 structure, in complex with a substrate mimic inhibitor (C8E4, **LIT-01**), was taken from the Protein Data Bank (PDB code: 4NRE; resolution: 2.63 Å).^6^ and prepared for the docking procedures using SYBYL-X^51^ v.2.1. All hydrogen atoms were added, and the protonation states of the ionizable amino acid side-chain groups were set at pH = 7.4. The side-chain amide groups of Asn and Gln residues were oriented in the direction of maximal potential hydrogen bonding. The cocrystallized ligand molecule (substrate mimic inhibitor) and all water molecules, except for those that coordinate iron, were removed from the original pdb file. The two water molecules that coordinate iron were considered for docking procedures, as described below. The catalytic iron and two calcium ions were kept as part of the protein structure. An energy minimization of the protein structure in successive stages was performed with the standard TRIPOS force field, using Powell’s method^99^ with simplex initial optimization, gradient termination of 0.05 kcal.mol^–1^.Å^–1^, and the maximum of iterations set to 500.

### Compounds Database Preparation

The structures of compounds from the ZINC-12^34,35^ database (∼23 × 10^6^ compounds) were downloaded in sdf format. Compounds from this database were submitted to a preliminary “curation” step, using the following criteria: (i) compounds should have good predicted water solubilities based on their calculated “Solubility Forecast Index” (SFI)^70^ values (compounds with SFI values ≤5.0 were considered to have a good predicted water solubility); and (ii) compounds should match the physicochemical and structural properties criteria defined according to the “drug” filter^100^ implemented in FILTER^101^ v.2.1.1 (with some modifications), which include: not violating more than two of the Lipinki’s Rule of Five^74,75^ criteria; having at least 15 non-hydrogen atoms; having 2–20 “heteroatoms” (noncarbon, non-hydrogen atoms); having a maximum of four chiral centers; having a maximum of 20 rotatable bonds; not having “promiscuous” protein-reactive groups^68,102^ (*e.g.*, quinones, aldehydes, Michael acceptors, epoxides, acid halides *etc.*); among others. Compounds that passed the filter criteria (∼8 × 10^6^) compose a database termed here “ZINC-Curated”. Compounds from the ZINC-Curated database were prepared for the VS procedures as follows: (i) the most abundant tautomeric forms and protonated states at pH = 7.4 were obtained using MoKa^92^ v.2.6; and (ii) up to 30 conformations per compound were generated using OMEGA^65−67^ v.2.5.1, with default parameters.

### Shape-Based Model Generation and Screening Procedures

Two known h15-LOX-2 inhibitors reported in the literature (referred to as MLS000545091 (**LIT-04**) and MLS000536924 (**LIT-05**), Figure 2)^28^ were used to generate a shape-based model (“query”), using ROCS^39,97^ v.3.3.0.3/v.3.4.3.0. The 3D structures of the two inhibitors were obtained in sdf format using Open Babel^87^ v.2.3.1 and, subsequently, the most abundant tautomeric form and protonation states of each compound at pH = 7.4 were obtained using the *Tautomers* and *FixpKa* tools from QUACPAC^96^ v.2.0.0.3. Next, a low energy conformation of each inhibitor was obtained using OMEGA^65−67^ v.2.5.1.5. Finally, the two compounds were aligned, using LigandScout’s^59^ v.4.4.7 alignment algorithm. The aligned compounds were used to generate a shape-based model (“query”) in ROCS, and default settings were used to run a shape-based screening in ROCS. As described in the literature,^39^ ROCS performs overlays of conformers of the candidate molecule to the generated “query” based on their shape-matching. For each compound in the database, the conformer that best matched the model was ranked according to its Shape Tanimoto^39^ score value, which is computed based on the shape overlap and ranges from 0 to 1.

### 2D Structural “Dissimilarity”^53^ Filter

To eliminate compounds that showed a high degree of structural similarity among each other, compounds were first clustered by 2D structural similarity, using molecular fingerprints, which are binary sets (“bits”) that encode the presence/absence of a certain substructure/fragment in a molecule.^53^ Default FP2 path-based fingerprints were generated using the Babel program.^87^ Based on the generated fingerprints, Tanimoto’s coefficient^103^ (TC) value was calculated for each pair of compounds. The “dissimilarity” indices (DI) were then calculated from Tanimoto’s coefficient values, as follows
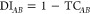
in which: DI*_AB_*: dissimilarity index for two molecules, *A* and *B*. TC*_AB_*: Tanimoto’s coefficient index for two molecules, *A* and *B*.

The calculated DI values were transferred to a data matrix, which was used as an input file in the R program^104^ for grouping structurally similar compounds into clusters, using a hierarchical clustering algorithm with complete linkage and the *hclust* function. Next, a DI cutoff value of 0.15 was applied, which means that compounds showing more than 85% 2D similarity to each other were grouped into the same cluster. Only one representative compound of each cluster was maintained for the next VS steps.

### Docking and Visual Inspection Procedures

Docking was performed using GOLD^54^ v.5.2. The h15-LOX-2 structure (PDB code: 4NRE; resolution: 2.63 Å) was prepared as described above and used for the docking calculations. The active site’s cavity, which was taken as the binding site region for docking, was defined by the residues Phe-184, Glu-369, His-373, His-378, Leu-415, Leu-420, Val-426, Leu-605, Ala-606, Leu-609, Leu-610, Ile-676, and by the catalytic iron. Two water molecules, which coordinate the protein’s catalytic iron, were allowed to switch on and off (*i.e*., protein-bound or displaced by the ligand) according to GOLD’s estimations of free-energy changes associated with moving a water molecule from the bulk solvent to the protein’s binding site. Ten docking runs were performed for each ligand, using default settings for genetic algorithm parameters, and ChemPLP^55^ and Goldscore^54^ as scoring functions. The docking procedure was validated by “redocking” the substrate mimic inhibitor C8E4 (**LIT-01**, cocrystallized with h15-LOX-2)^6^ into the active site’s cavity, using the same settings as described above, except for not considering the two coordinating water molecules. A careful visual inspection analysis of the best-scored pose of each docked compound was performed, using LigandScout^59^ v.4.4.7 and PyMOL^95^ v.2.1.0, to check the fit into h15-LOX-2 active site’s cavity. In this analysis, the overall quality of the binding was evaluated based on recognition of the main protein–ligand interactions and on the analysis of shape complementarity between the compound and the protein’s binding site. Docking poses that showed unrealistic, sterically hindered conformations were excluded.

### h15-LOX-2 Expression and Purification

*E. coli* BL21 DE3 pLysS cells were transformed with pETDuet-1 containing the gene encoding His-tagged h15-LOX-2 (50–60 ng) by electroporation. After transformation, cells grew up as cultures in LB broth (Sigma-Aldrich) containing ampicillin (100 μg·mL^–1^) (Sigma-Aldrich) and chloramphenicol (170 μg·mL^–1^) (Sigma-Aldrich), at 37 °C, for ∼20h, at 150–190 rpm, and were subsequently diluted with 500 mL of LB medium to enough concentration to initiate cell growth with an OD_600_ = 0.2. Cells were maintained at 37 °C under agitation until the exponential growth phase was reached (OD_600nm_ = 0.6–1.0). Gene expression was then induced with isopropyl-β-D-thiogalactopyranoside (IPTG, 0.5 mM) (Sigma-Aldrich), in LB broth, for 12 h, at 20 °C, under agitation (170 rpm). Afterward, cells were centrifuged (5000 rpm, 4 °C, 15 min) and resuspended in Tris buffer (50 mM, pH = 8,0) containing NaCl (500 mM), lysozyme (50 μg·mL^–1^), and protease inhibitor (1× Sigma FAST Protease Inhibitor Tablet). Cells were lysed in an ice bath by sonication for 5 min (40% amplitude, alternating between 20 s of sonication and 60 s of rest). Next, streptomycin (1% w/w) (Sigma-Aldrich) was added, and the lysate was maintained in ice by 15 min, under agitation. The lysate was then centrifuged (14,000 rpm, 4 °C, 40 min), and soluble proteins were filtered using a Durapore 0.45 μm PVDF filter (Merck, Germany).

h15-LOX-2 was purified by affinity chromatography (Ni^2+^-NTA Superflow, 1 mL) (Qiagen, Netherlands), using the ÄKTA Protein Purification system (Amersham Pharmacia Biotech). The column was first washed (“equilibrated”) with 10 mL of buffer A (Tris buffer (50 mM, pH = 8) and NaCl (500 mM)), with a flow rate of 1 mL·min^–1^. Purification was performed in four steps, with a flow rate of 1 mL·min^–1^, and the protein was eluted with a 5–100% gradient of “Buffer B” (Tris buffer (50 mM, pH = 8), NaCl (500 mM) and imidazole (500 mM)). Fractions were analyzed by SDS-PAGE and those containing h15-LOX-2 were pooled and concentrated with an Amicon Ultra Centrifugal Filter (30 kDa cutoff) (Merck, Germany). Finally, a HiTrap 5 mL Desalting (Cytiva) column was used for removing imidazole. The concentration of purified h15-LOX-2 was estimated by absorbance measurements at λ = 280 nm (ε = 115,445 M^–1^·cm^–1^),^105^ at room temperature, in a quartz cuvette.

### Enzymatic Assays

All enzymatic assays were carried out in quartz cuvettes (path length of 1 cm), in a total volume of 300 μL, containing Tris buffer (25 mM, pH = 8.0), NaCl (250 mM), h15-LOX-2 (120–420 nM), Triton X-100 (0.014% v/v), arachidonic acid (25 μM), and the tested compound (1.0 μM to up to 199.1 μM, depending on the compound solubility in the assay buffer). The system was preincubated at room temperature for 2 min with all reagents, except for the substrate (arachidonic acid). Next, the enzyme reaction was started by adding arachidonic acid. The enzyme reaction was monitored spectrophotometrically, following the formation of the product (15(*S*)-HpETE, ε = 25,000 M^–1^·cm^–1^)^106^ at λ = 234 nm, for 20–100 s, at room temperature. Stock solutions of the tested compounds were prepared in DMSO, and the concentration of DMSO in the assays was 1.0% (v/v). Each assay was performed at least in duplicates. Data analyses were carried out using the program GraphPad Prism v.9. A four-parameter logistic nonlinear regression model was used to build the concentration–response curves and to obtain the IC_50_ values. A mixed-type inhibition model was used to calculate *K_i_* values after inhibition-type diagnosis based on Lineweaver–Burk plots. All reagents used for enzymatic assays were obtained from Sigma-Aldrich, except for Triton X-100, which was obtained from Vetec (Brazil).

### Purity Statement

Compounds tested as inhibitors (**1** – **14**) were purchased from MolPort (*Molport SIA*, Riga, Latvia) and are >88% pure. The purities of Compounds **07**, **10**, **11**, **12**, **13**, and **14**, which were identified as h15-LOX-2 inhibitors, were determined by ^1^H qNMR, using DMSO-*d*_6_ as a solvent and maleic acid as an internal calibrant, following the journal’s guidelines (see Table S9). These compounds are at least 88% pure. ^1^NMR or liquid chromatography–mass spectrometry (LC-MS) spectra for **1** – **14** are available in Supporting Information.

### Turbidimetric Assays for Solubility Estimations

The solubilities of compounds that showed inhibitory activities higher than 10% at 10 μM were estimated by turbidimetric assays in the same conditions used for the enzymatic assays described above. With this purpose, samples of each compound at different concentrations (5–500 μM) were prepared in a solution containing Tris buffer (25 mM, pH = 8.0), NaCl (250 mM), and Triton X-100 (0.014% v/v). Stock solutions of each compound were prepared in DMSO, and the final DMSO concentration in each analyzed sample was 1.0% (v/v). Turbidity (light scattering) of each sample was measured at λ = 500 nm, considering that the tested compounds had absorbance peaks below this wavelength. Measurements were done using a *Hitachi* U-2010 spectrophotometer.

## Supporting Information Available

Validation of the shape-based model; validation of docking procedures; descriptive statistical parameters for scores of compounds docked into h15-LOX-2; structures and physicochemical properties of compounds selected by VS; ChemPLP and Goldscore score values for compounds selected and not selected by the visual inspection filter; SwissADME predictions of physicochemical properties and drug-likeness for compounds **1**–**14**; representative results of h15-LOX-2 expression/purification; Michaelis–Menten plot and kinetic parameters for h15-LOX-2-catalyzed reaction; effects of DMSO and of Triton X-100 on h15-LOX-2 activity; concentration–response curve for NDGA (**LIT-02**); Turbidimetric assays for solubility estimation; schematic representation of mixed-type inhibition model; Inhibition kinetics for compounds **10** and **13**; score values of the identified h15-LOX-2 inhibitors (compounds **07**, **10**, **11**, **12**, **13**, and **14**) docked into h15-LOX-2, h5-LOX, h12-LOX, and rabbit reticulocyte h15-LOX-1 active sites; comparison of the predicted binding modes of compounds **10** and **13** into the h15-LOX-2 active site’s cavity and into h15-LOX-2’s allosteric binding site; representation of SZMAP and GamePlan calculations results; ^1^H NMR and/or LC-MS spectra for compounds **1**–**14** (data provided by suppliers); data used to calculate the purities of compounds **07**, **10**, **11**, **12**, **13**, and **14** (PDF)
Molecular strings and biological data for compounds **1**–**14** (CSV)
Docking poses for compounds **07**, **10**, **11**, **12**, **13**, and **14** (ZIP)

## Abbreviations Used

15(*S*)-HpETE: 15S-hydroperoxy-(5Z,8Z,11Z,13E)-eicosatetraenoate
C8E4: octyltetraethylene glycol ether
COXs: cyclooxygenases
DI: “dissimilarity” index
DMSO: dimethyl sulfoxide
h15-LOX-2: human 15-lipoxygenase-2
IPTG: isopropyl-β-d-thiogalactopyranoside
LB: Luria–Bertani broth
LC-MS: liquid chromatography coupled to mass spectrometry
LOXs: lipoxygenases
NDGA: nordihydroguaiaretic acid
Ni^2+^-NTA: nickel-nitrilotriacetic acid
^1^H NMR: proton nuclear magnetic resonance
OD_600_: optical density at λ = 600 nm
PAINS: pan assay interference compounds
PDB: Protein Data Bank
PEBP1: phosphatidylethanolamine (PE)-binding protein 1
PLAT: polycystin-1, lipoxygenase, α-toxin
PVDF: polyvinylidene fluoride
RMSD: root-mean-square deviation
ROCS: rapid overlay of chemical structures
SDS-PAGE: sodium dodecyl sulfate–polyacrylamide gel electrophoresis
SEM: standard error of the mean
SFI: solubility forecast index
TC: Tanimoto’s coefficient
VS: virtual screening
