# Supplementary material for: Identification of Novel Human 15-Lipoxygenase-2 (h15-LOX-2) Inhibitors Using a Virtual Screening Approach
Source: J Med Chem. 2024 Dec 19;68(1):307–23. doi: 10.1021/acs.jmedchem.4c01884 (PMC11726667; doi:10.1021/acs.jmedchem.4c01884)
Supplement: Supplementary file 1 — jm4c01884_si_001.pdf [file jm4c01884_si_001.pdf]

SUPPORTING INFORMATION

Identification of novel human 15-lipoxygenase-2 (h15-LOX-2) inhibitors using a virtual screening approach

Lucas G. Viviani[1†], Thais S. Iijima[1†], Erika Piccirillo[2], Leandro Rezende[1], Thiago G. P. Alegria[3], Luis Eduardo S. Netto[3], Antonia T.-do Amaral[4], and Sayuri Miyamoto[1*]*

[1] Department of Biochemistry, Institute of Chemistry, University of São Paulo, Av. Prof. Lineu Prestes 748, 05508-000 São Paulo, Brazil.

[2] Center for Medicinal Chemistry (CQMED), State University of Campinas, Av. André Tosello 550, 13083-886 Campinas, Brazil.

[3] Department of Genetics and Evolutionary Biology, Institute of Biosciences, University of São Paulo, Rua do Matão, 277, 05508-090 São Paulo, Brazil.

[4] Department of Fundamental Chemistry, Institute of Chemistry, University of São Paulo, Av. Prof. Lineu Prestes 748, 05508-000 São Paulo, Brazil.

[*] Corresponding authors: lucas.viviani@usp.br, miyamoto@iq.usp.br.

[†] These authors contributed equally to this work.

Table of Contents

Content	Page(s)
Validation of the shape-based model (**Figure S1**)	S3
Validation of docking procedures (**Tables S1** and **S2**; **Figures S2** and **S3**)	S4 – S5
Descriptive statistic parameters for scores of compounds docked into h15-LOX-2 (**Table S3**)	S6
Structures and physicochemical properties for compounds selected by VS (**Table S4**)	S7 – S13
ChemPLP and Goldscore score values for compounds selected and not selected by the visual inspection filter (**Table S5**)	S14
SwissADME predictions of physicochemical properties and drug-likeness for compounds **1** – **14** (**Figure S4**)	S15
Representative results of the expression and purification of h15-LOX-2 (**Figure S5**)	S16
Michaelis-Menten plot for the h15-LOX-2-catalyzed reaction (**Figure S6)**	S16
Kinetic parameters calculated for h15-LOX-2-catalyzed reaction (**Table S6**)	S17
Effect of DMSO on the h15-LOX-2 activity (**Figure S7**)	S17
Effect of Triton X-100 on the h15-LOX-2 activity (**Figure S8**)	S18
Concentration-response curve for NDGA (**LIT-02**) (**Figure S9**)	S18
Turbidimetric assays for solubility estimation (**Table S7**)	S19
Schematic representation of mixed-type inhibition model (**Scheme S1**)	S20
Inhibition kinetics for compounds **10** and **13** (**Figure S10**)	S21
Score values of the identified h15-LOX-2 inhibitors (compounds **07**, **10**, **11**, **12**, **13**, and **14**) docked into h15-LOX-2, h5-LOX, h12-LOX, and rabbit reticulocyte h15-LOX-1 active sites (**Table S8**)	S22
Comparison of the predicted binding modes of compounds **10** and **13** into the h15-LOX-2 active site's cavity and into h15-LOX-2's allosteric binding site (**Figure S11**)	S23 – S24
Representation of SZMAP and GamePlan calculations results (**Figure S12**)	S25
^{1}H NMR or LC-MS spectra for compounds **1** – **14**	S26 – S39
Data used to calculate the purities of compounds **07**, **10**, **11**, **12**, **13**, and **14** (**Table S9**)	S40
References	S41

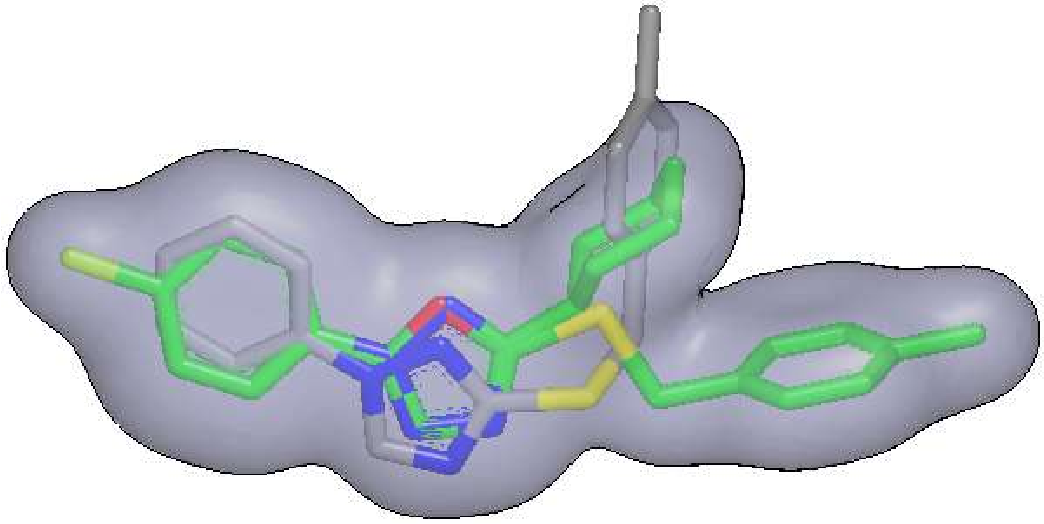

Figure S1. Superposition of **LIT-05** (represented as sticks, carbon atoms in gray), in the conformation it is observed in the crystal structure of the complex with h15-LOX-2 (PDB code: 7LAF; resolution: 2.44 Å),[1] with the generated shape-based model (represented as a gray surface). The structures of **LIT-04** and **LIT-05**, in the respective low energy conformations that were used to build the shape-based model, are represented as sticks (carbon atoms in green). Oxygen, nitrogen, sulfur, and chlorine are shown in red, blue, yellow, and light green, respectively. The figure was generated using ROCS.

Table S1. RMSD values calculated between the pose of the substrate mimic inhibitor C8E4 (**LIT-01**) observed in the crystal structure (PDB code: 4NRE; resolution: 2.63 Å)[2] and the two best-scored docking poses (poses 1 and 2).

**Pose (ranked)**	**RMSD values(*) (Å)**			
	**ASP**	**ChemPLP**	**ChemScore**	**GoldScore**
1	2.65	2.75	7.06	2.07
2	4.02	2.82	6.93	3.89
Average (1 and 2)	3.34	2.79	7.00	2.98

(*) Calculated between the docking pose and the pose observed in the crystal structure (PDB code: 4NRE; resolution: 2.63 Å).[2]

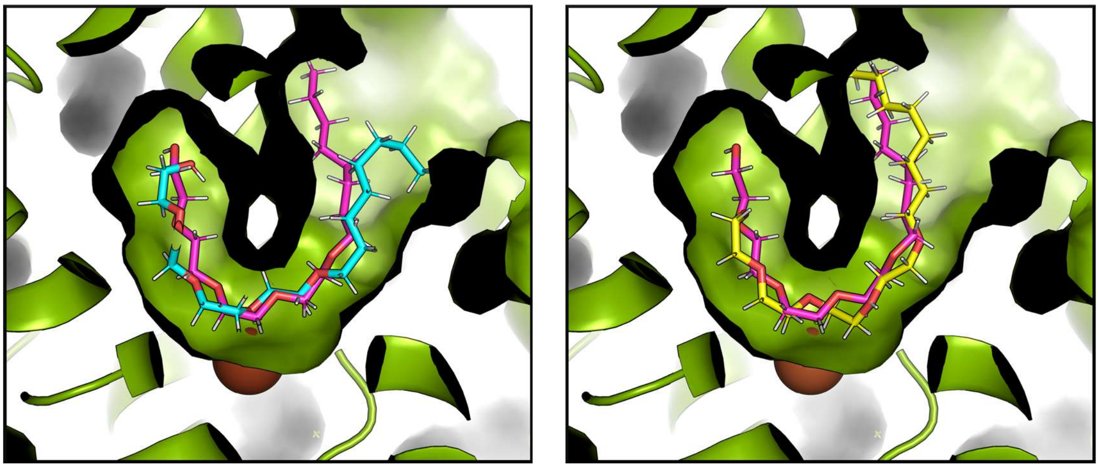

Figure S2. Superposition between the crystallographic pose observed for the inhibitor C8E4 in the 15-LOX-2-C8E4 complex (PDB code: 4NRE[2], resolution: 2.63 Å, magenta sticks) and the respective best-scored docking solutions (pose 1) obtained using ChemPLP (cyan sticks, left) and GoldScore (yellow sticks, right) scoring functions. The catalytic iron is represented as a brown sphere. The figure was prepared using PyMOL.

Table S2. RMSD values calculated between the pose of the inhibitor **LIT-05** observed in the crystal structure (PDB code: 7LAF; resolution: 2.44 Å)[1] and the two best-scored docking poses (poses 1 and 2).

Pose (ranked)	RMSD values(*) (Å)			
	**ASP**	**ChemPLP**	**ChemScore**	**GoldScore**
1	5.63	4.56	8.63	3.63
2	1.59	6.08	6.61	6.58
Average (1 and 2)	3.61	5.32	7.62	5.11

(*) Calculated between the docking pose and the pose observed in the crystal structure (PDB code: 7LAF, resolution: 2.44 Å).[1]

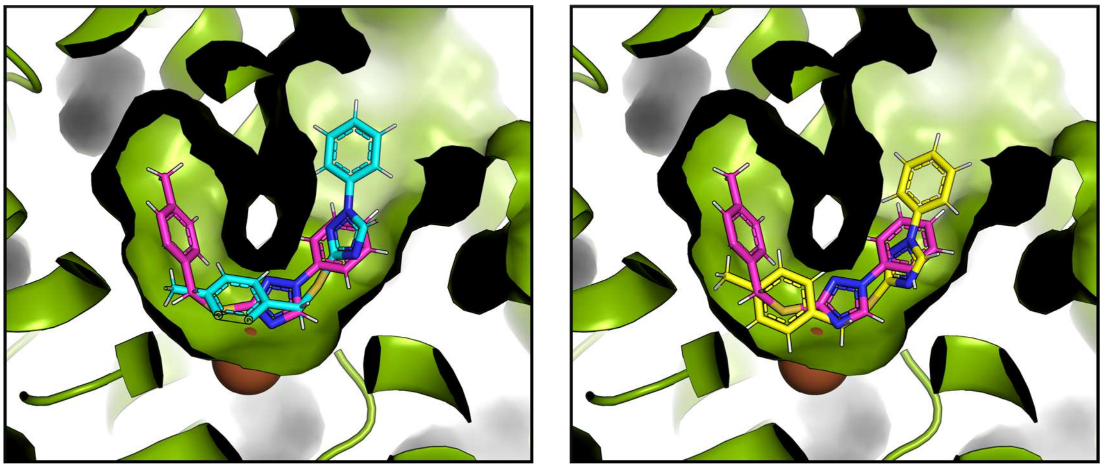

Figure S3. Superposition between the crystallographic pose observed for the inhibitor **LIT-05** in the 15-LOX-2-**LIT-05** complex (PDB code: 7LAF[1], resolution: 2.44 Å, magenta sticks) and the respective best-scored docking solutions (pose 1) obtained using ChemPLP (cyan sticks, left) and GoldScore (yellow sticks, right) scoring functions. The catalytic iron is represented as a brown sphere. The figure was prepared using PyMOL.

Table S3. Descriptive statistic parameters for the score values of compounds docked into h15-LOX-2 using ChemPLP and Goldscore scoring functions.[(*)]

	ChemPLP	Goldscore
**Minimum**	53.68	5.76
**25% Percentile**	70.59	57.60
**Median**	74.64	63.37
**75% Percentile**	78.99	67.62
**Maximum**	98.34	84.24
**Range**	44.66	78.48
**Mean**	74.79	61.74
**Std. Deviation**	6.46	9.34
**Std. Error of Mean**	0.29	0.42

[(*)] Calculated using GraphPad Prism 10.2.2.

Table S4. Structures and physicochemical properties of the compounds selected as potential h15-LOX-2 inhibitors from the ZINC-Curated database by the proposed VS protocol.

Ref. and/or ZINC ID	Structure[(a)]	Molecular formula	Molecular weight ($g.mol^{-1}$)	LogP[(b)]	Number of HBA[(c)]	Number of HBD[(c)]	Number of rotatable bonds[(c)]	TPSA[(c)] ($Å^2$)
**01** ZINC63152202	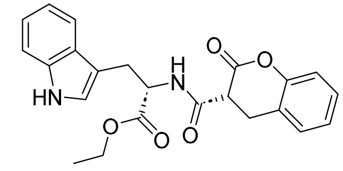	$C_{23}H_{22}N_2O_5$	406.4	0.69	6	1	9	97.55
**02** ZINC64045009	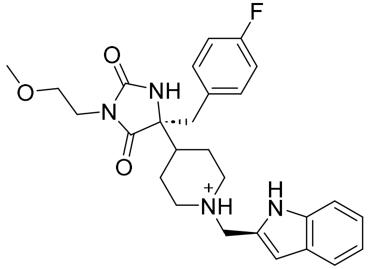	$C_{27}H_{31}FN_4O_3$	479.6	1.5	5	2	9	74.24
**03** ZINC14989654	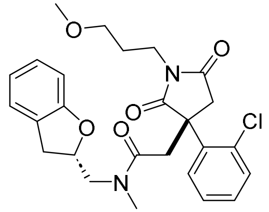	$C_{26}H_{29}ClN_2O_5$	485.0	3.2	5	0	11	76.15
**04** ZINC21018084	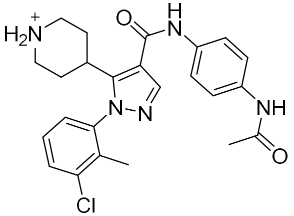	$C_{24}H_{26}ClN_5O_2$	453.0	4.5	3	4	7	92.63
**05** ZINC63667415	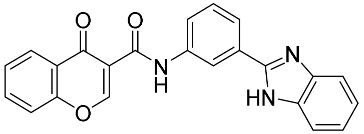	$C_{23}H_{15}N_3O_3$	381.4	3.0	4	1	3	84.03

Table S4. (*Continued*)

**06** ZINC09445447	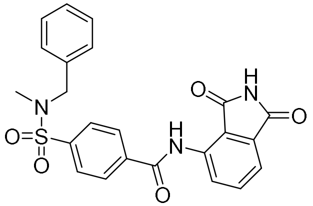	$C_{23}H_{19}N_3O_5S$	449.5	2.6	5	2	7	112.65
**07** ZINC10187184	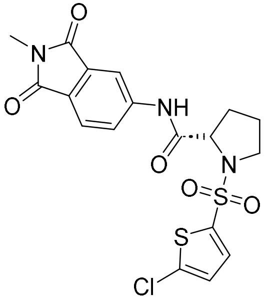	$C_{18}H_{16}ClN_3O_5S_2$	453.9	2.4	5	1	5	103.86
**08** ZINC17148759	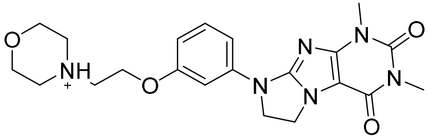	$C_{21}H_{26}N_6O_4$	426.5	0.30	5	0	7	86.76
**09** ZINC23138421	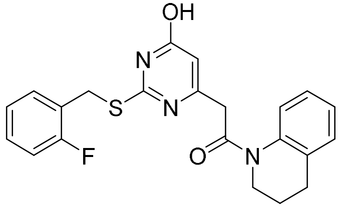	$C_{22}H_{20}FN_3O_2S$	409.5	4.1	5	1	5	66.06
**10** ZINC00794703	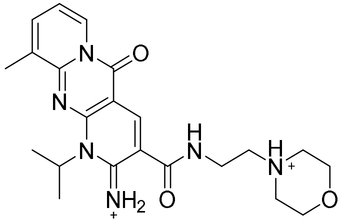	$C_{22}H_{28}N_6O_3$	425.5	1.1	4	2	8	106.46

Table S4. (*Continued*)

**11**<br>ZINC02395677	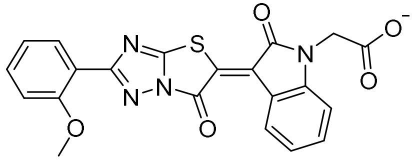	$C_{21}H_{14}N_4O_5S$	433.4	1.2	7	0	5	116.93
**12**<br>ZINC63362107	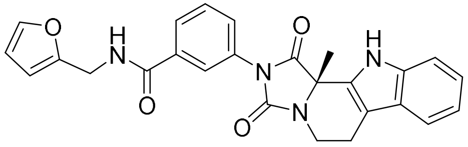	$C_{26}H_{22}N_4O_4$	454.5	2.0	4	1	5	95.22
**13**<br>ZINC18202958	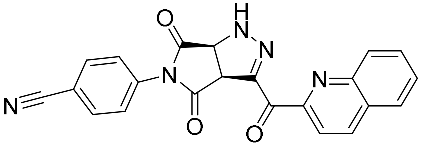	$C_{22}H_{13}N_5O_3$	395.4	1.8	6	1	3	115.52
**14**<br>ZINC32124366	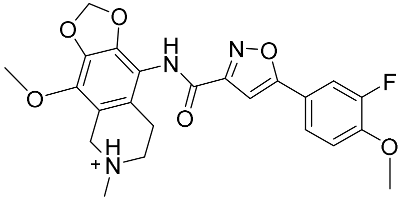	$C_{23}H_{22}FN_3O_6$	456.5	3.5	6	2	8	95.29
ZINC79045996	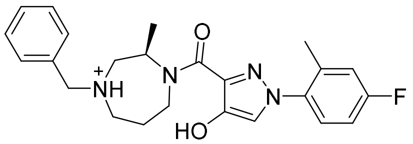	$C_{24}H_{27}FN_4O_2$	423.5	3.8	4	2	7	61.6
ZINC77939079	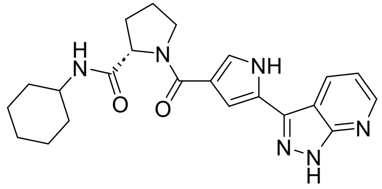	$C_{22}H_{26}N_6O_2$	406.5	3.0	4	3	4	106.77
ZINC14012965	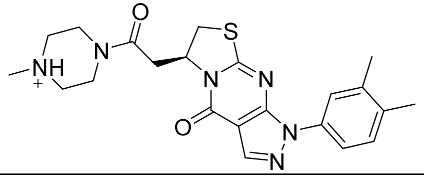	$C_{22}H_{26}N_6O_2S$	439.6	2.0	5	1	6	76.26

Table S4. (*Continued*)

ZINC39733003	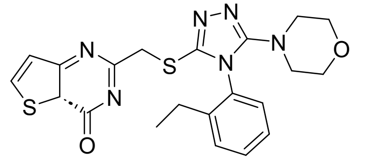	$C_{21}H_{22}N_6O_2S_2$	454.6	2.8	8	0	7	84.97
ZINC64622398	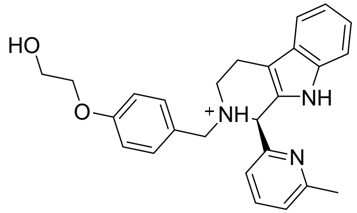	$C_{26}H_{27}N_3O_2$	414.5	2.6	4	2	8	57.95
ZINC13109340	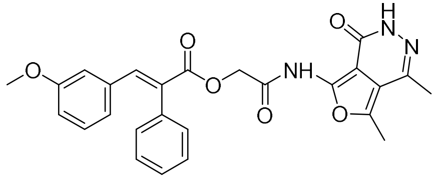	$C_{26}H_{23}N_3O_6$	473.5	3.9	5	2	11	123.52
ZINC33010296	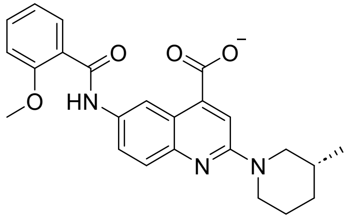	$C_{24}H_{25}N_3O_4$	418.5	4.4	5	1	7	94.59
ZINC33260851	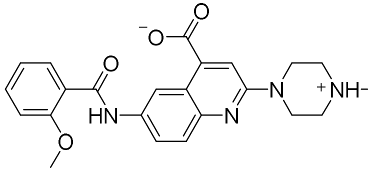	$C_{23}H_{24}N_4O_4$	420.5	2.9	5	2	7	97.83
ZINC15269234	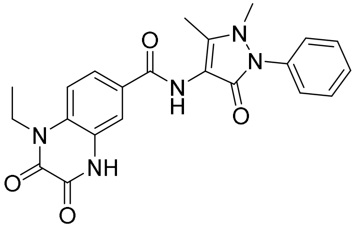	$C_{22}H_{21}N_5O_4$	419.4	1.8	4	2	7	110.89

Table S4. (*Continued*)

ZINC72465538	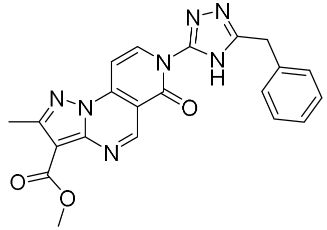	$C_{21}H_{17}N_7O_3$	414.4	1.8	7	0	7	117.16
ZINC19418460	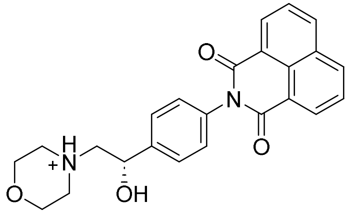	$C_{24}H_{22}N_2O_4$	402.5	3.0	4	1	5	70.08
ZINC09120398	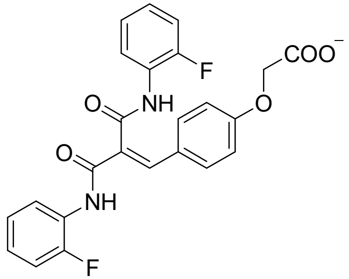	$C_{24}H_{18}F_2N_2O_5$	451.4	4.1	7	2	8	107.56
ZINC39467137	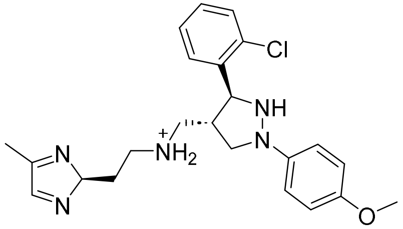	$C_{23}H_{28}ClN_5O$	427.0	3.9	3	2	10	65.83
ZINC02323981	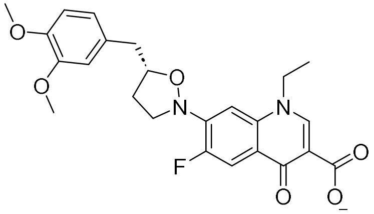	$C_{24}H_{25}FN_2O_6$	455.5	3.6	7	0	10	93.06

Table S4. (*Continued*)

ZINC11785568	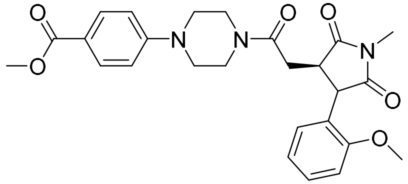	$C_{26}H_{29}N_3O_6$	479.5	1.8	5	0	10	96.46
ZINC67554839	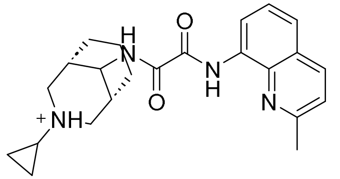	$C_{23}H_{28}N_4O_2$	393.5	2.9	3	3	5	74.33
ZINC20792548	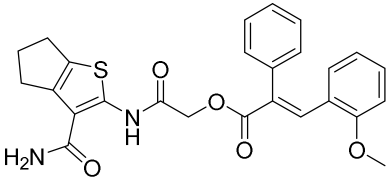	$C_{26}H_{24}N_2O_5S$	476.6	4.1	4	2	10	107.72
ZINC63667383	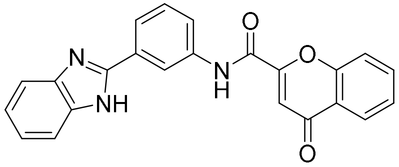	$C_{23}H_{15}N_3O_3$	381.4	3.0	4	1	3	84.03
ZINC64007007	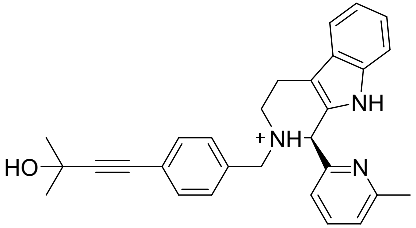	$C_{29}H_{29}N_3O$	436.6	3.3	3	2	5	48.72
ZINC12035964	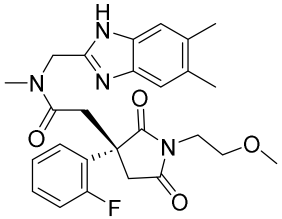	$C_{26}H_{29}FN_4O_4$	480.5	3.0	6	1	12	95.6

Table S4. (*Continued*)

ZINC11783806	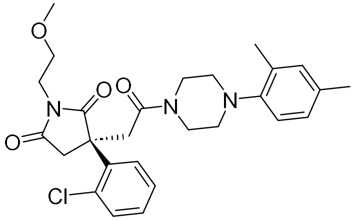	$C_{27}H_{32}ClN_3O_4$	498.0	3.3	4	0	10	70.16
ZINC64031717	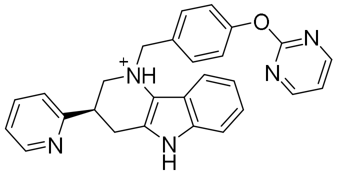	$C_{27}H_{23}N_5O$	434.5	3.5	4	1	5	63.5
ZINC69046714	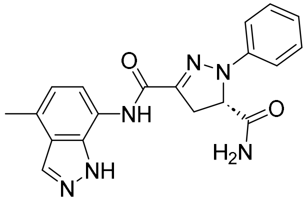	$C_{19}H_{18}N_6O_2$	362.4	1.9	4	3	5	116.47
ZINC38631038	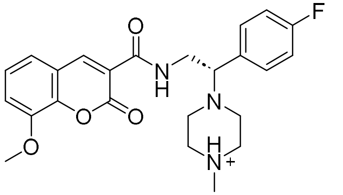	$C_{24}H_{26}FN_3O_4$	440.5	2.7	4	2	8	75.02
ZINC12037832	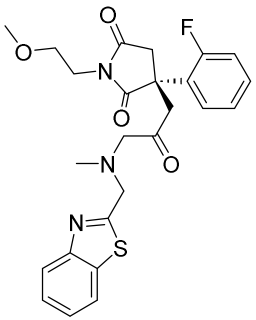	$C_{24}H_{24}FN_3O_4S$	469.5	3.1	6	0	10	79.81

[(a)] Structures are represented considering the most abundant protonation states for ionizable groups at pH = 7.4, according to MoKa and/or FixpKa predictions for pK_a.

[(b)] LogP values were taken from the ZINC database.

[(c)] Number of hydrogen-bond acceptors (HBA), number of hydrogen-bond donors (HBD), number of rotatable bonds, and topological polar surface area (TPSA) values were calculated using LigandScout.

Table S5. ChemPLP and Goldscore score values for compounds selected and not selected by the visual inspection filter.

ChemPLP scores		Goldscore scores	
**Selected (16)**	**Not selected (34)**	**Selected (30)**	**Not selected (20)**
83.42	86.57	74.11	74.55
84.98	84.84	72.42	75.09
90.66	85.88	74.7	72.56
83.69	86.72	74.07	77.83
85.24	84.18	84.24	73.63
90.65	85.08	72.87	71.73
87.69	88.33	74.62	72.75
90.06	86.26	73.26	74.09
84.84	87.15	72.77	75.46
89.4	90.12	72.65	74.94
98.34	84.9	72.92	74.36
91.56	84.19	73.2	73.47
89.96	85.61	78.32	75.22
89.13	84.1	72.48	73.14
84.49	87.04	72.1	72.35
95.87	84.21	76.54	71.99
	83.23	73.91	73.19
	86.26	74.45	71.86
	84.02	73.35	73.19
	85.21	73.87	73.87
	83.62	81.05	
	85.91	81.63	
	83.36	74.17	
	84.05	78.95	
	83.56	76.49	
	84.68	74.14	
	83.75	75.79	
	86.52	77.96	
	83.76	72.12	
	83.8	79.71	
	84.69		
	83.7		
	85.05		
	84.8		
**88.75**(*)	**85.15**(*)	**75.29**(*)	**73.76**(*)

(*) Average values.

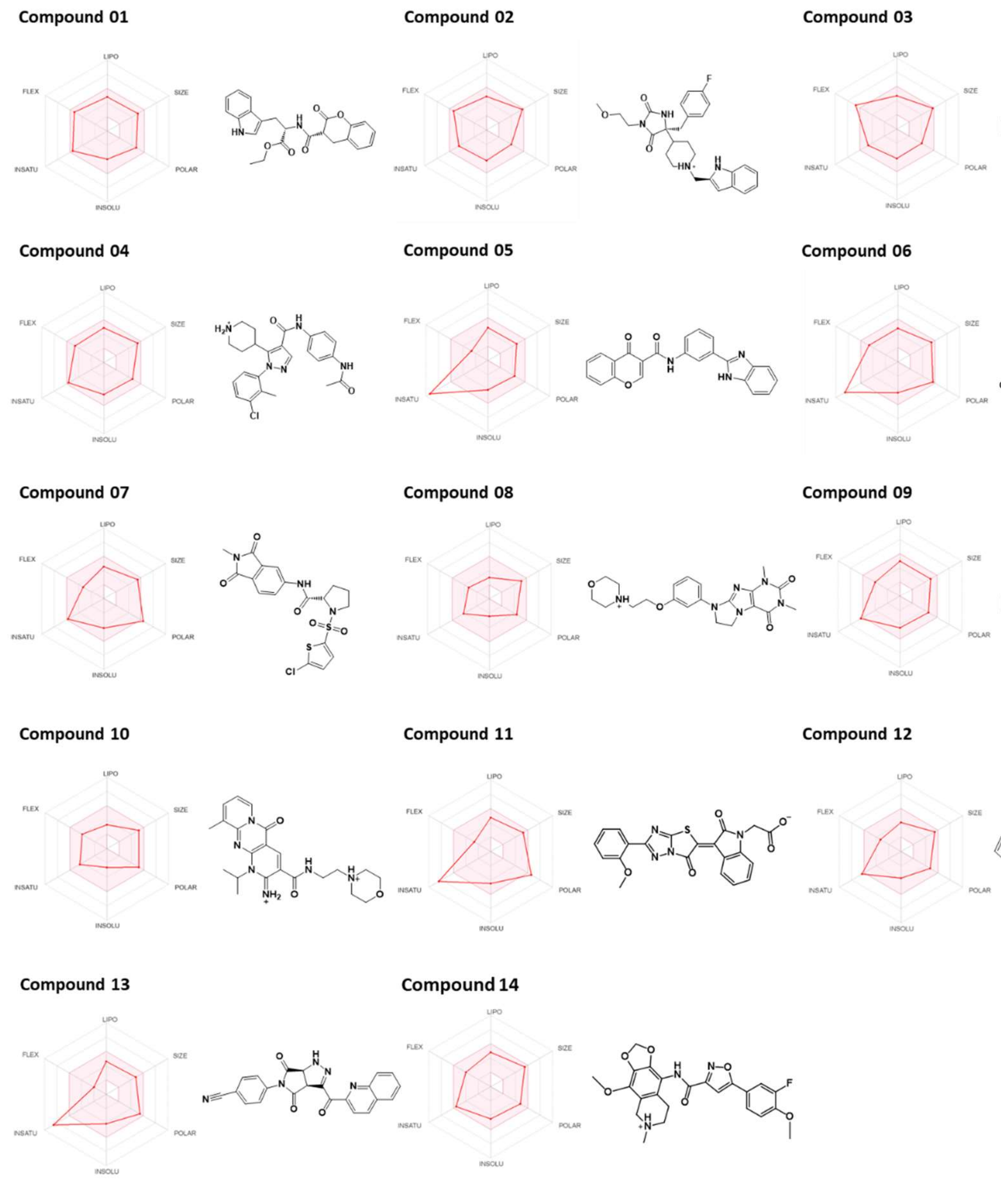

Figure S4. SwissADME[3] "bioavailability radar" plots for the compounds selected as h15-LOX-2 inhibitor candidates from the ZINC-Curated database and that have been acquired for enzymatic inhibitory assays. The colored zone in the diagrams represents the suitable physicochemical space for oral bioavailability, according to the following parameter limits: LIPO (lipophilicity): XLOGP3 between −0.7 and +5.0; SIZE: molecular weight between 150 and 500 g.mol^{-1}; POLAR (polarity): TPSA between 20 and 130 Å^2; INSOLU (solubility): LogS lower than 6; INSATU (degree of saturation): fraction of carbons with sp^3 hybridization between 0.25 and 1.0; and FLEX (flexibility): no more than 9 rotatable bonds.

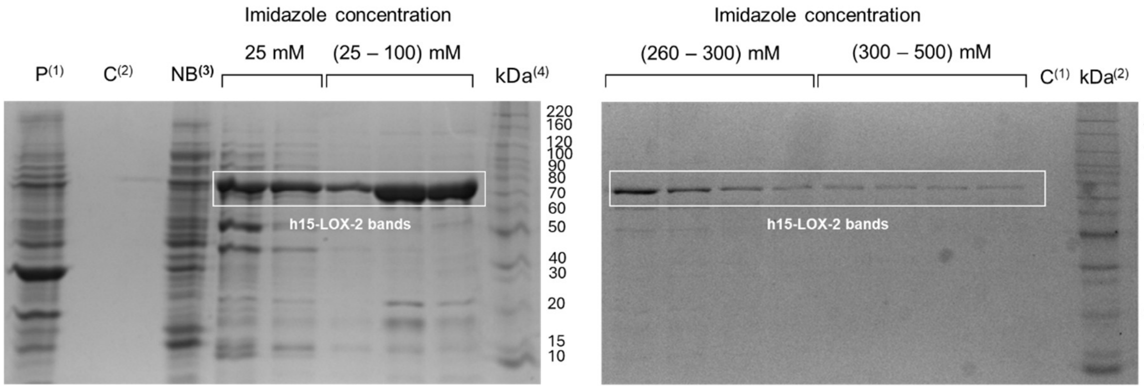

Figure S5. Representative results of the expression and purification of h15-LOX-2 (SDS-PAGE analysis of the collected peaks). (1) P: Cell pellet; (2) C: Control (loading buffer); (3) NB: Lysate supernatant not-bound to the Ni^{2+}-NTA affinity column; (4) Molecular weight markers: BenchMark™ Protein Ladder (*Invitrogen*).

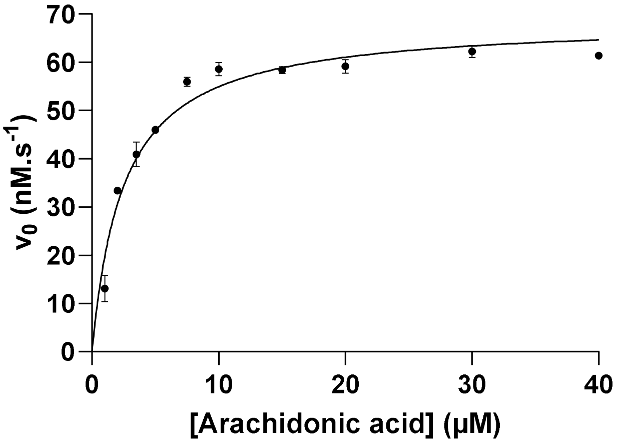

Figure S6. Plot of initial velocities of h15-LOX-2-catalyzed reaction as a function of arachidonic acid concentrations. A Michaelis-Menten model was used to fit the experimental data. Assays were carried out in a total volume of 300 µL, containing Tris buffer (25 mM, pH = 8.0), NaCl (250 mM), h15-LOX-2 (120 nM), Triton X-100 (0.017% v/v), and arachidonic acid (1.0 - 40 µM). Data represent the average ± S.E.M. of experiments performed twice and at least in duplicates. The figure was prepared using GraphPad Prism.

Table S6. Kinetic parameters calculated for h15-LOX-2-catalyzed reaction, using arachidonic acid as a substrate.[(a)]

Kinetic parameter	Experimentally obtained value	Reference value (literature)[(b)]
K_M (μM)	2.5 ± 0.3	1.9 ± 0.37
V_{max} ($nM{\cdot}s^{-1}$)	68.7 ± 1.8	-
k_{cat} (s^{-1})	0.57	0.6 ± 0.02

(a) Data represent the averages from two independent experiments performed in duplicates. Assays were carried out in a total volume of 300 μL, containing Tris buffer (25 mM, pH = 8.0), NaCl (250 mM), h15-LOX-2 (120 nM), Triton X-100 (0.017% v/v), and arachidonic acid (1.0 - 40 μM).

(b) Reference: Kobe et al., *J. Biol. Chem.*, 289, 12, 2014.

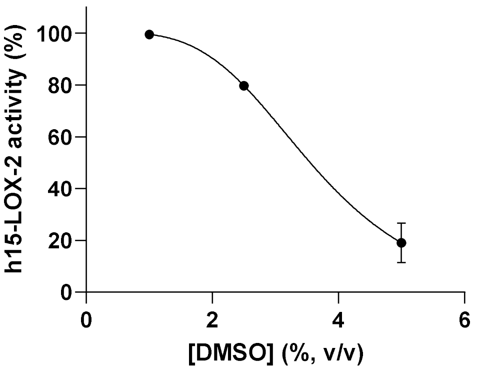

Figure S7. Effect of DMSO on the h15-LOX-2 activity. Assays were carried out in a total volume of 300 μL, containing Tris buffer (25 mM, pH = 8.0), NaCl (250 mM), h15-LOX-2 (120 nM), Triton X-100 (0.01% v/v), arachidonic acid (25 μM), and DMSO (0 - 5.0%, v/v). Data represent the average ± S.E.M. of experiments performed in duplicates. The figure was prepared using GraphPad Prism.

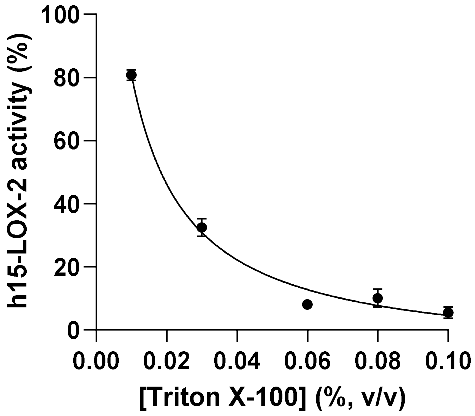

Figure S8. Effect of Triton X-100 on the h15-LOX-2 activity. Assays were carried out in a total volume of 300 μL, containing Tris buffer (25 mM, pH = 8.0), NaCl (250 mM), h15-LOX-2 (420 nM), arachidonic acid (25 μM), and Triton X-100 (0 – 0.1% v/v). Data represent the average ± S.E.M. of experiments performed in duplicates. The figure was prepared using GraphPad Prism.

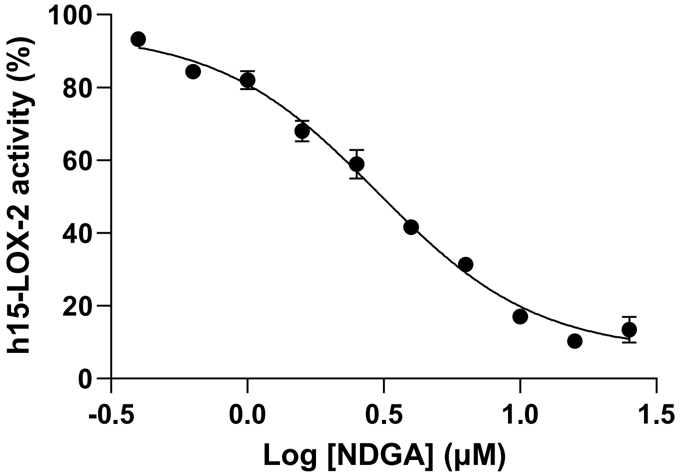

Figure S9. Concentration-response curve for NDGA (LIT-02). All assays were carried out in a total volume of 300 μL, containing Tris buffer (25 mM, pH = 8.0), NaCl (250 mM), h15-LOX-2 (120 nM), Triton X-100 (0.014% v/v), arachidonic acid (25 μM), and NDGA (0.4 μM – 25.1 μM). Data represent the average ± S.E.M. of two independent experiments, each performed in duplicates. The Hill slope value for the concentration-response curve is of 1.5 ± 0.2. The figure was prepared using GraphPad Prism.

Table S7. Turbidity values at different concentrations and estimated solubilities for compounds **07**, **10**, **11**, **12**, **13**, and **14**.[a]

	Turbidity (at λ = 500 nm)[b]					
**Concentration (µM)**	**Compound 07**	**Compound 10**	**Compound 11**	**Compound 12**	**Compound 13**	**Compound 14**
500	0.32 ± 0.02	0.000	-	0.12 ± 0.03	0.32 ± 0.03	-
315.5	0.29 ± 0.01	-	-	0.3715 ± 0.0005	0.11 ± 0.04	0.281 ± 0.002
250	-	-	0.028	-	-	-
200	-	-	0.024	-	-	-
199	0.239 ± 0.009	-	-	0.355	0.002 ± 0.002	-
125.6	0.157 ± 0.005	-	-	0.302	0.0005 ± 0.0005	-
125	-	-	0.01 ± 0.01	-	-	0.11 ± 0.01
80	-	-	-	-	-	0.08 ± 0.02
100	-	-	0.0005 ± 0.0005	-	-	-
79.2	0.04 ± 0.01	-	-	0.231	-	-
62.5	-	-	-	-	-	0.007
50	0.001 ± 0.001	-	-	0.004	-	0.007
31.5	0	-	-	0.008	-	0.005
19.9	0.005 ± 0.001	-	-	0.007	-	0
12.6	0.005 ± 0.004	-	-	0.002	-	0
7.3	0.01	-	-	0	-	0.002
5	0.009	-	-	0.001	-	0.002
**Estimated solubility (S) (range, µM)[c]**	50 ≤ S < 79.2	S ≥ 500	100 ≤ S < 125	12.6 ≤ S < 19.9	199 ≤ S < 315.5	32 < S < 50
**LogP[d]**	2.4	1.1	1.2	2	1.8	3.5
**Concentration range in the enzymatic assays (µM)[e]**	1.0 - 39.8	3.2 - 199.5	1.6 - 100	1.0 - 15.8	2.0 - 125.6	-

[a] Assays were carried out in Tris buffer (25 mM, pH = 8.0), NaCl (250 mM), and Triton X-100 (0.014% v/v). The DMSO concentration in each sample was 1.0% (v/v).
[b] Values represent the average of duplicates.
[c] Solubilities were estimated considering a cutoff value of 0.005 for turbidity (highlighted areas in the table represent values above this cutoff).
[d] LogP values were taken from the ZINC database.
[e] Concentration ranges used for obtaining the concentration-response curves in the enzymatic assays.

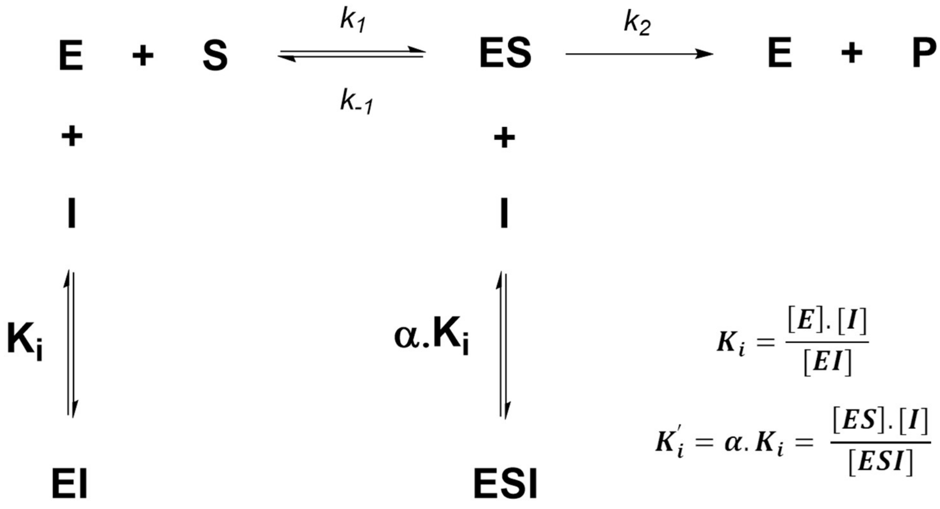

Scheme S1. Schematic representation of a simplified, well-accepted model for equilibria between enzyme, substrate, enzyme-substrate complex, a mixed-type inhibitor, enzyme-inhibitor complex, and enzyme-substrate-inhibitor complex. E: free enzyme; S: substrate; P: product; I: inhibitor; ES: enzyme-substrate complex; EI: enzyme-inhibitor complex; ESI: enzyme-substrate-inhibitor complex; k_1: kinetic constant of ES formation; k_{-1}: kinetic constant of ES breakdown; k_2: kinetic constant for P formation; K_i: equilibrium constant for inhibitor binding to E; K_i': equilibrium constant for inhibitor binding to ES.

Modified from Burker, Boriack-Sjodin, and Copeland.[4]

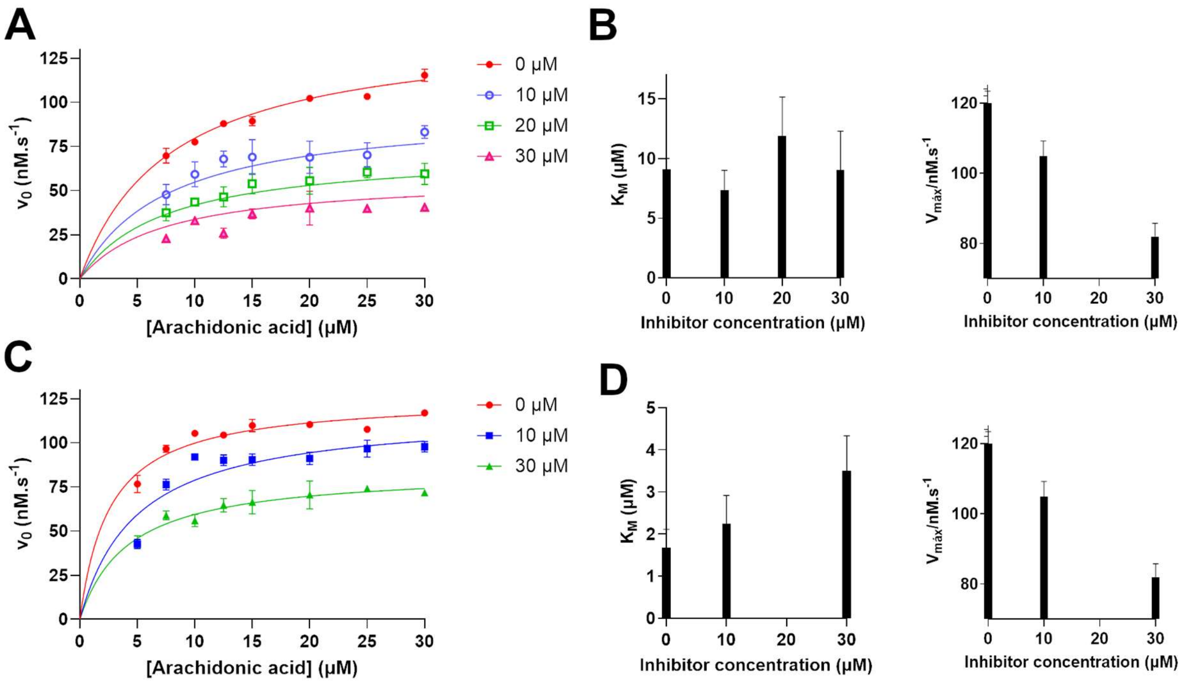

Figure S10. Inhibition kinetics for compounds **10** and **13.** (A) Michaelis-Menten plots in the absence of inhibitor and in the presence of compound **10** at 10 µM, at 20 µM, and at 30 µM. (B) K_M and V_{max} values obtained from the Michaelis-Menten plots for compound **10**. (C) Michaelis-Menten plots in the absence of inhibitor and in the presence of compound **13** at 10 µM and at 30 µM. (B) K_M and V_{max} values obtained from the Michaelis-Menten plots for compound **13**.

Table S8. Score values of the identified h15-LOX-2 inhibitors (compounds **07**, **10**, **11**, **12**, **13**, and **14**) docked into h15-LOX-2, h5-LOX, h12-LOX, and rabbit reticulocyte h15-LOX-1 active sites.

Compound (ID)	Compound (ZINC ID)	h15-LOX-2		h5-LOX[(a)]		h12-LOX[(b)]		Rabbit 15-LOX-1[(c)]	
		ChemPLP	Goldscore	ChemPLP	Goldscore	ChemPLP	Goldscore	ChemPLP	Goldscore
**07**	ZINC10187184	76.81	72.48	54.27	55.83	64.42	54.89	61.80	62.24
**10**	ZINC00794703	79.70	78.32	21.14	-72.84	57.59	21.44	63.74	62.12
**11**	ZINC02395677	63.18	78.95	17.83	40.56	49.66	35.13	64.13	63.10
**12**	ZINC63362107	83.69	72.65	32.04	14.56	50.68	17.64	75.77	60.87
**13**	ZINC18202958	70.39	74.07	22.00	45.08	60.64	53.70	63.38	62.87
**14**	ZINC32124366	66.73	72.12	10.87	0.00	56.98	52.53	64.00	59.88

(a) PDB code: 7TTK; resolution: 1.98 A; 42.23% sequence identity with h15-LOX-2.

(b) PDB code: 8GHB; resolution: 2.76 A; 36.82% sequence identity with h15-LOX-2.

(c) PDB code: 1LOX; resolution: 2.40 A; 80.97% sequence identity with h15-LOX-1; 37.28% sequence identity with h15-LOX-2.

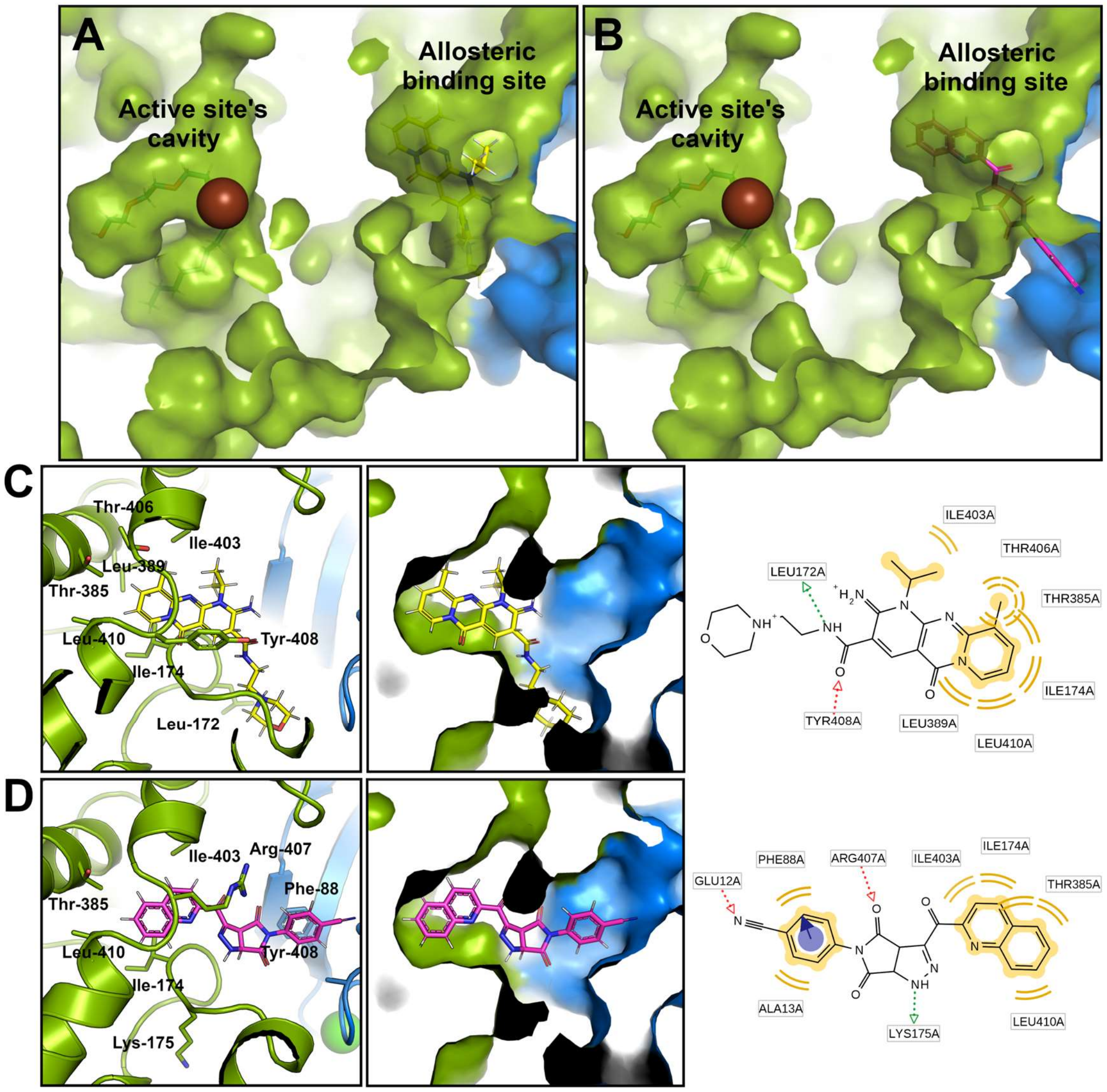

Figure S11. Schematic representations of the predicted binding modes (best-scored docking solutions) of compounds **10** and **13** into the h15-LOX-2's allosteric binding site and of the binding mode of the substrate mimic inhibitor C8E4 (**LIT-01**) into the h15-LOX-2 active site's cavity (crystallographic pose reported in the literature).[2]

(A, B) Overview of the h15-LOX-2 active site's cavity and of the h15-LOX-2 allosteric binding site, showing the predicted binding modes of compounds **10** (**A**, carbon atoms shown in yellow) and **13** (**B**, carbon atoms shown in pink) into the allosteric binding site. The structure of the co-crystallized inhibitor C8E4 (**LIT-01**, carbon atoms shown in light blue), which binds the h15-LOX-2 active site's cavity as a substrate mimic,[2] is represented to model the binding of **10** or **13** to the h15-LOX-2-substrate complex. h15-LOX-2's C- and N-terminal domains are represented as green and blue surfaces, respectively. The catalytic iron is shown as a brown sphere.

(C, D) Close-up view of the allosteric binding site, showing the predicted binding mode of compounds **10** (**C**) and **13** (**D**). *Left panels*: Schematic representations of the best-scored docking solutions for each inhibitor inside the h15-LOX-2's allosteric binding site. For clarity, only the residues that interact with each inhibitor are highlighted (shown as sticks). *Central panels*: Schematic representations of the best-scored docking solutions for each compound, with h15-LOX-2 structure represented as a surface. *Right panels*: 2D Schematic representations of the protein-ligand interactions identified using the LigandScout program.

Figure S10. (***Continued***) Yellow: hydrophobic interactions; blue circle/arrow: pi-cation interactions; red arrows: hydrogen-bond acceptor interactions; green arrows: hydrogen-bond donor interactions.
The figure was prepared using PyMOL.

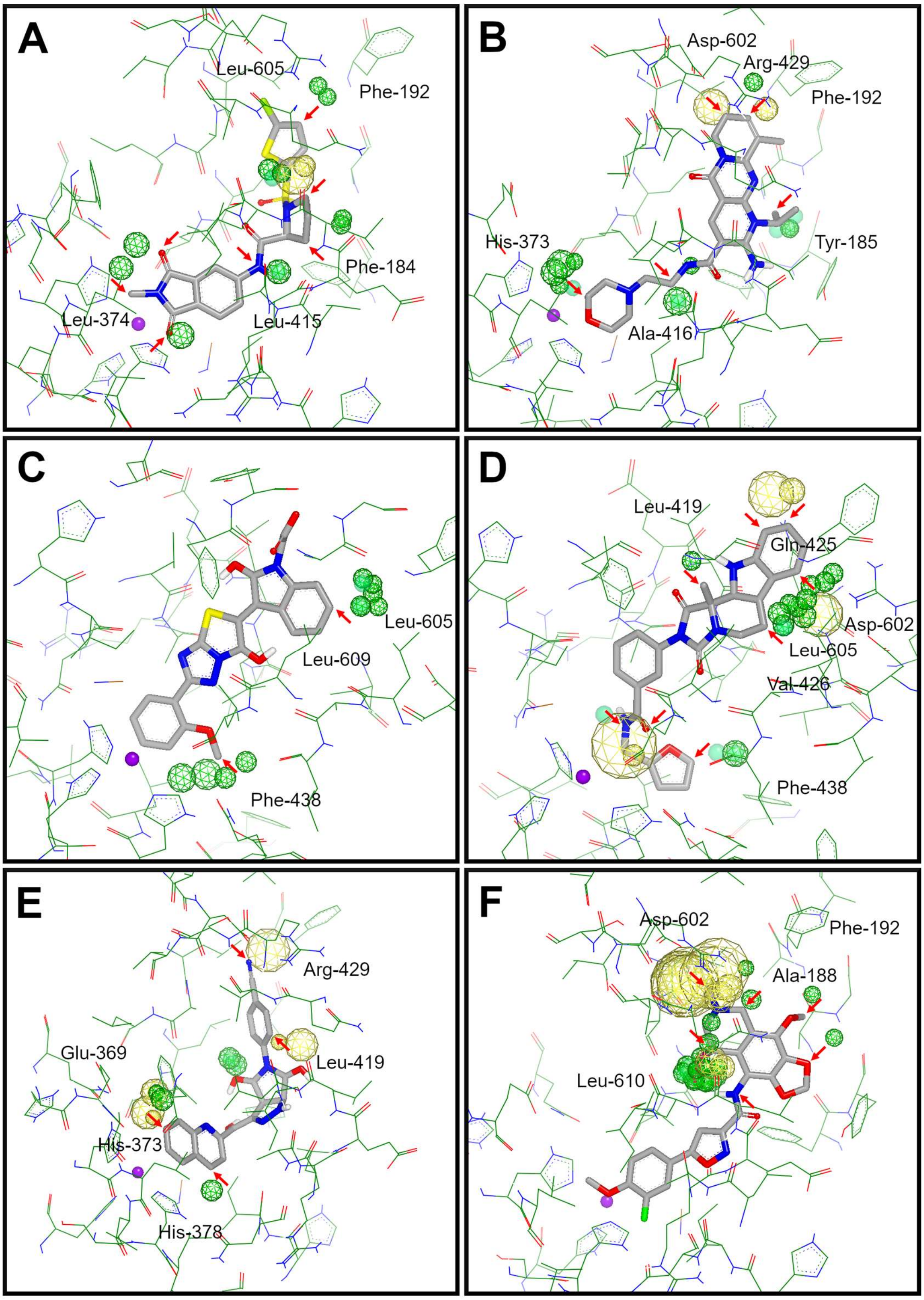

Figure S12. Representation of SZMAP and GamePlan calculations results. Red arrows indicate points in the ligand structure where attachment of a substituent (or replacement by another substituent/group) would increase binding affinity. "Polar sites" (yellow mesh spheres) and "van der Waals sites" (green mesh spheres) indicate regions that might be occupied by substituents able to make polar and van der Waals interactions. Structures of the inhibitors are represented as sticks: **(A)** Compound **07**; **(B)** Compound **10**; **(C)** Compound 11; **(D)** Compound **12; (E)** Compound **13**; and **(F)** Compound **14**. Carbon, oxygen, nitrogen and sulfur atoms are colored in gray, red, blue and yellow, respectively. Protein residues are represented as lines. The iron ion are shown a purple sphere. The figure was prepared using VIDA.

^{1}NMR OR LC-MS SPECTRA FOR COMPOUNDS 1 – 14

(Data provided by suppliers/manufacturers)

<u>Compound 01</u>

(ChemBridge Corporation. ID: 5677507)

^{1}H NMR Spectrum **(provided by the supplier)**

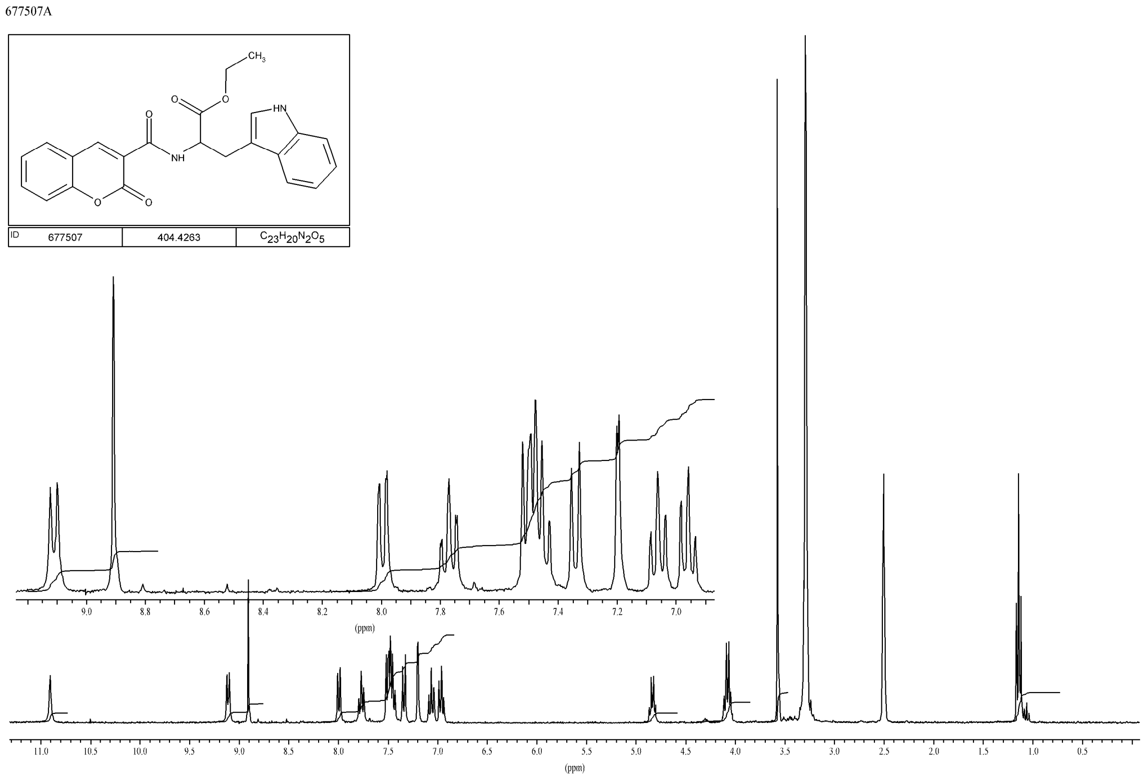

Compound 02

(ChemBridge Corporation. ID: 22020451)

LC-MS Spectrum (provided by the supplier)

ST400125

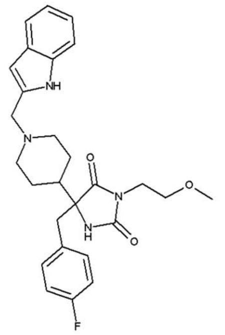

ID	22020451	478.5715	$C_{27}H_{31}FN_4O_3$

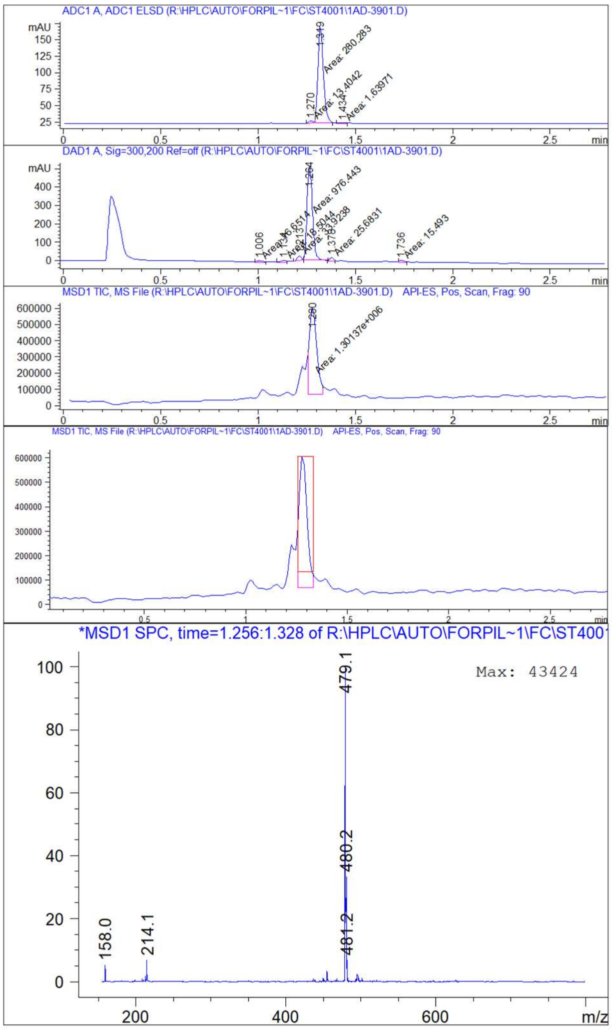


```
Data File R:\HPLC\AUTO\FORPIL~1\FC\ST4001\1AD-3901.D
Sample Name: ST4001P1-A-04
Instrument 1 31/08/2017 12:37:07  4
Column: ONYX MONOLITHIC, C18 50x4.6mm |3.75ml/min| Columns Reg
Valve
Gradient: "A"->@2.0min->"B"(Hold 0.6min)->@0.2min->"A"->PostRun
PMP1, Solvent A         : 0.1TFA in AcN
PMP1, Solvent B         : 0.1TFA in 2.5%AcN/W
PMP1, Solvent C         : H2O
PMP1, Solvent D         : AcN
Ionization mode            :    API-ES Positive

Signal 1: ADC1 A, ADC1 ELSD
Peak RetTime Type  Width     Area      Height     Area
  #   [min]        [min]   [mAU*s]     [mAU]        %
----|-------|----|-------|----------|----------|--------|
   1   1.270 MM    0.0633   13.40422    3.52774   4.5388
   2   1.319 MM    0.0307  280.28253  151.95798  94.9060
   3   1.434 MM    0.0369    1.63971 7.40781e-1   0.5552
Totals :                   295.32647  156.22649


Signal 2: DAD1 A, Sig=300,200 Ref=off
Peak RetTime Type  Width     Area      Height     Area
  #   [min]        [min]   [mAU*s]     [mAU]        %
----|-------|----|-------|----------|----------|--------|
   1   1.006 MM    0.0407   16.65139    6.81668   1.5323
   2   1.134 MM    0.0361   18.50439    8.53903   1.7028
   3   1.213 MM    0.0227   33.92384   24.88758   3.1217
   4   1.264 MM    0.0315  976.44250  516.41193  89.8541
   5   1.378 MM    0.0228   25.68306   18.78042   2.3634
   6   1.736 MM    0.0289   15.49302    8.92441   1.4257
Totals :                  1086.69821  584.36004


Signal 3: MSD1 TIC, MS File
Peak RetTime Type  Width     Area      Height     Area
  #   [min]        [min]                            %
----|-------|----|-------|----------|----------|--------|
   1   1.280 MM    0.0395 1.30137e6  5.49187e5  100.0000
Totals :                  1.30137e6  5.49187e5
```

Compound 03

(ChemBridge Corporation. ID: 43312106)

LC-MS Spectrum **(provided by the supplier)**

ST201504

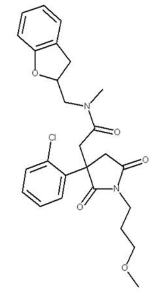

ID	43312106	484.9844	$C_{26}H_{29}ClN_2O_5$

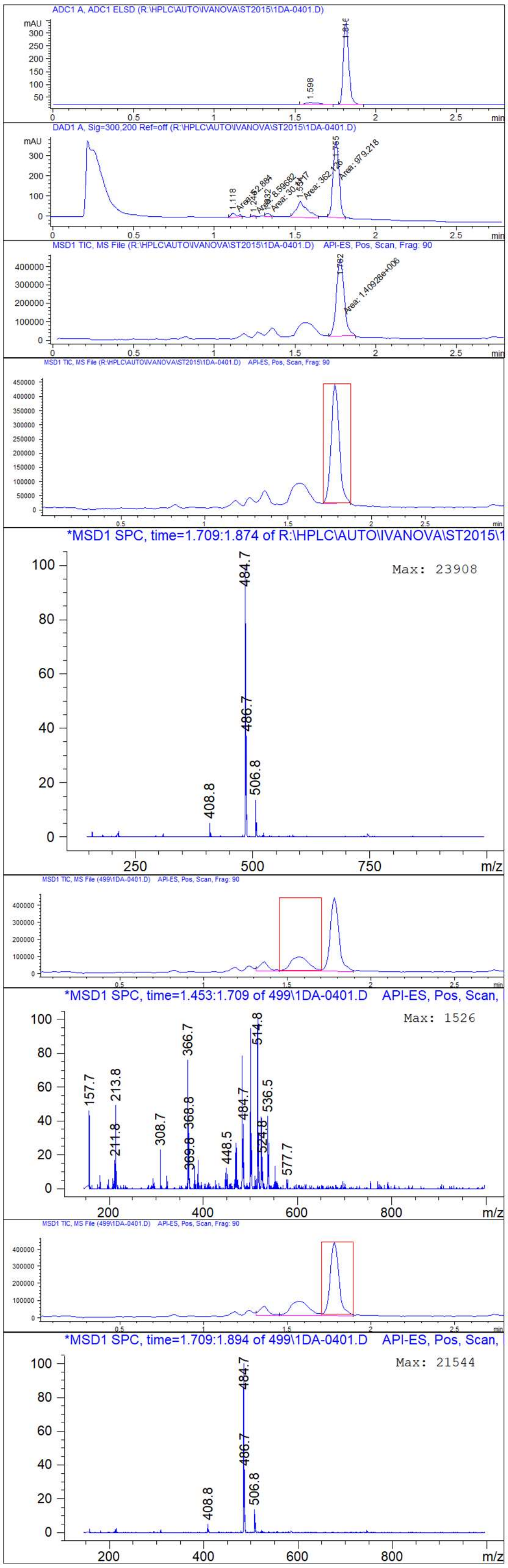


```
Data File R:\HPLC\AUTO\IVANOVA\ST2015\1DA-0401.D
Sample Name: ST2015P1-D-01
Instrument 1 25/03/2016 17:41:01
Column: Onyx C18 50x4.6mm | 3.75ml/min | Columns Reg Valve
Gradient: "A"->@2.2min->"B"(Hold 0.4min)->@0.2min->"A"->PostRun
PMP1, Solvent A          : 0.1TFA in AcN
PMP1, Solvent B          : 00.1TFA in 2.5%AcN/W
PMP1, Solvent C          :
PMP1, Solvent D          :
Ionization mode              :    API-ES Positive
```

Signal 1: ADC1 A, ADC1 ELSD

Peak #	RetTime [min]	Type	Width [min]	Area [mAU*s]	Height [mAU]	Area %
1	1.598	BB	0.0669	32.73033	6.42930	4.3460
2	1.816	BB	0.0362	720.38440	321.12289	95.6540
Totals :				753.11473	327.55220	

Signal 2: DAD1 A, Sig=300,200 Ref=off

Peak #	RetTime [min]	Type	Width [min]	Area [mAU*s]	Height [mAU]	Area %
1	1.118	MM	0.0416	52.88403	21.17476	3.6906
2	1.244	MM	0.0216	8.59682	6.63041	0.5999
3	1.332	MM	0.0310	30.11173	16.17836	2.1014
4	1.534	MM	0.0750	362.12598	80.49558	25.2716
5	1.755	MM	0.0433	979.21777	377.28262	68.3364
Totals :				1432.93633	501.76174	

Signal 3: MSD1 TIC, MS File

Peak #	RetTime [min]	Type	Width [min]	Area	Height	Area %
1	1.782	MM	0.0553	1.40928e6	4.24378e5	100.0000
Totals :				1.40928e6	4.24378e5	

Compound 04

(ChemDiv. ID: G362-0661)

[1]*H NMR Spectrum* (provided by the supplier)

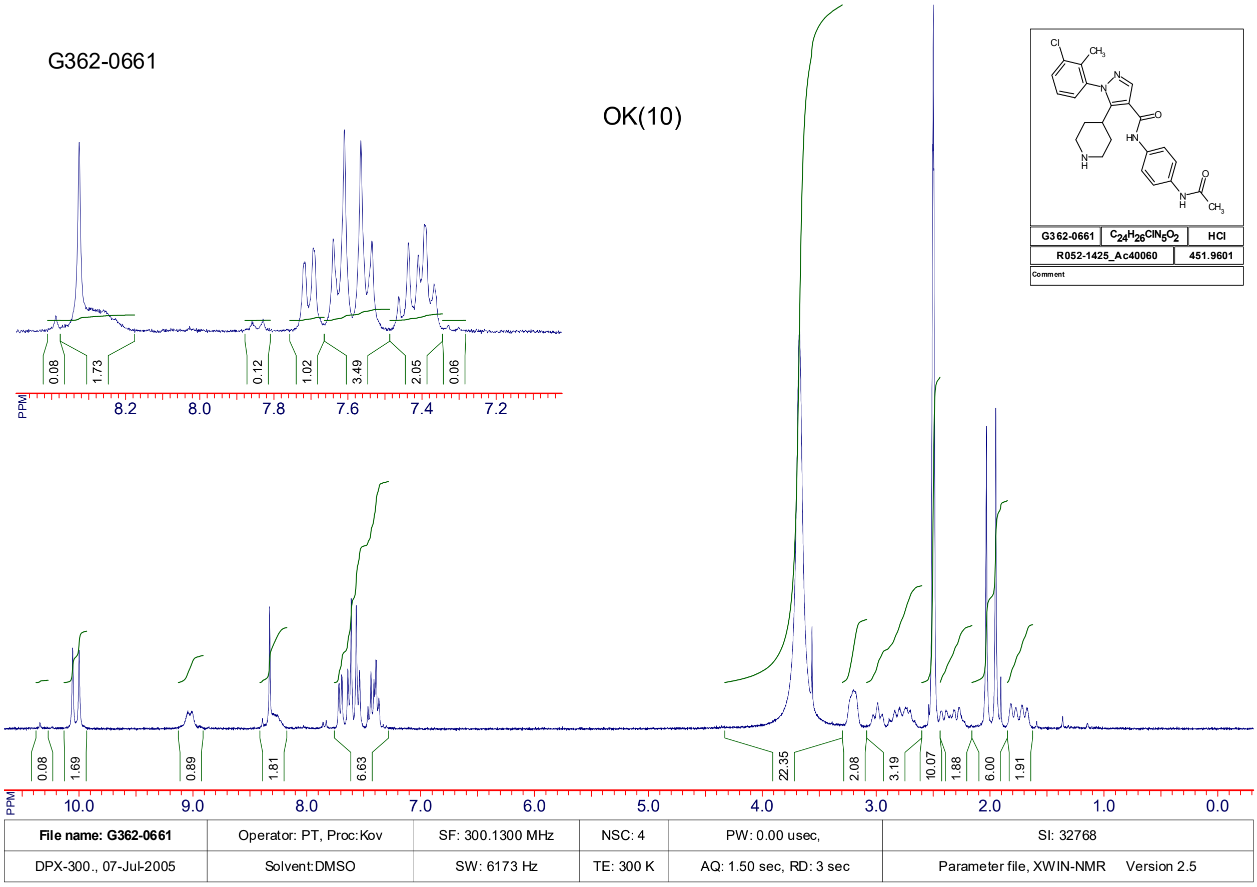

Compound 05

(Life Chemicals. ID: F0882-0549)

LC-MS Spectrum **(provided by the supplier)**

```
-.o.-Syntez Purity Report -.o.-
Agilent 1100 LC/MSD SL                               Mobile Phase:A-H2O+0.1%HCOOH;B-MeCN+0.1HCOOH
Diodearrey G1315B (DAD1A-215nm; DAD1B-254nm)         Separation column:
Mass Quad  G1956B (MSD1-Pos,    MSD2-Neg)            Rapid Resolutionn HT Cartige 4.6x30mm,
ELSD Altech 3300  (ADC1 A, ELSD)                     1.8-Micron, Zorbx SB-C18
```

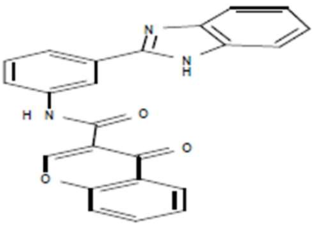

Mol.Weight: 381,39

F0882-0549

M10144 ->

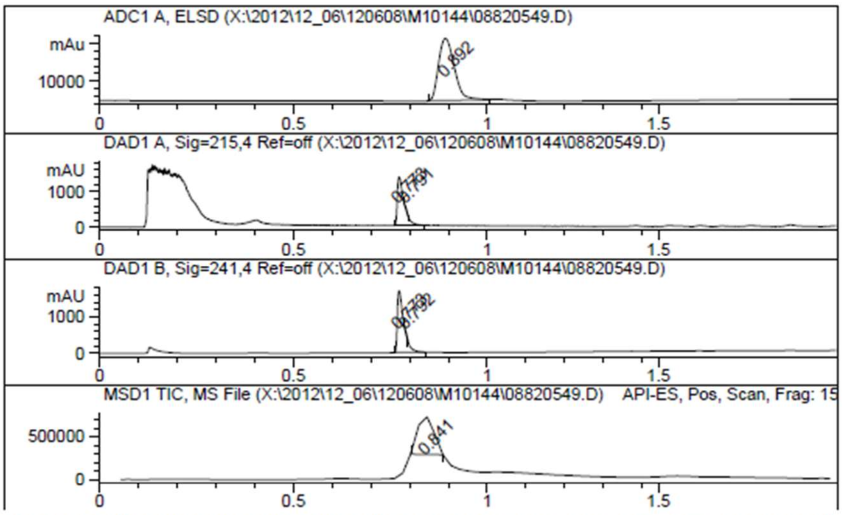

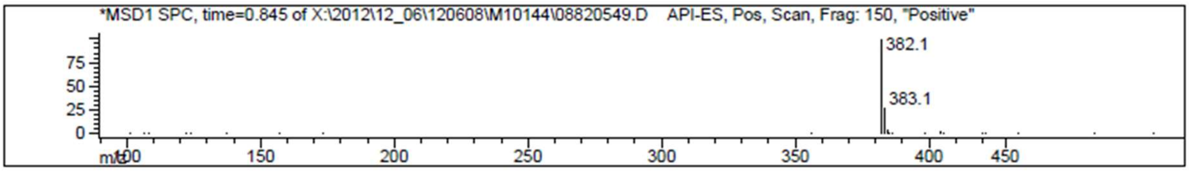

#	Signal	R.Time	Area %
1	ADC1 A, ELSD	0.892	100.000

#	Signal	R.Time	Area %
1	DAD1 A, Sig=215,4 Ref=off	0.773	99.060
2		0.791	0.940

#	Signal	R.Time	Area %
1	DAD1 B, Sig=241,4 Ref=off	0.773	99.075
2		0.792	0.925

#	Signal	R.Time	Area %
1	MSD1 TIC, MS File	0.841	100.000

Compound 06

(Life Chemicals. ID: F1120-1604)

^{1}H NMR Spectrum **(provided by the supplier)**

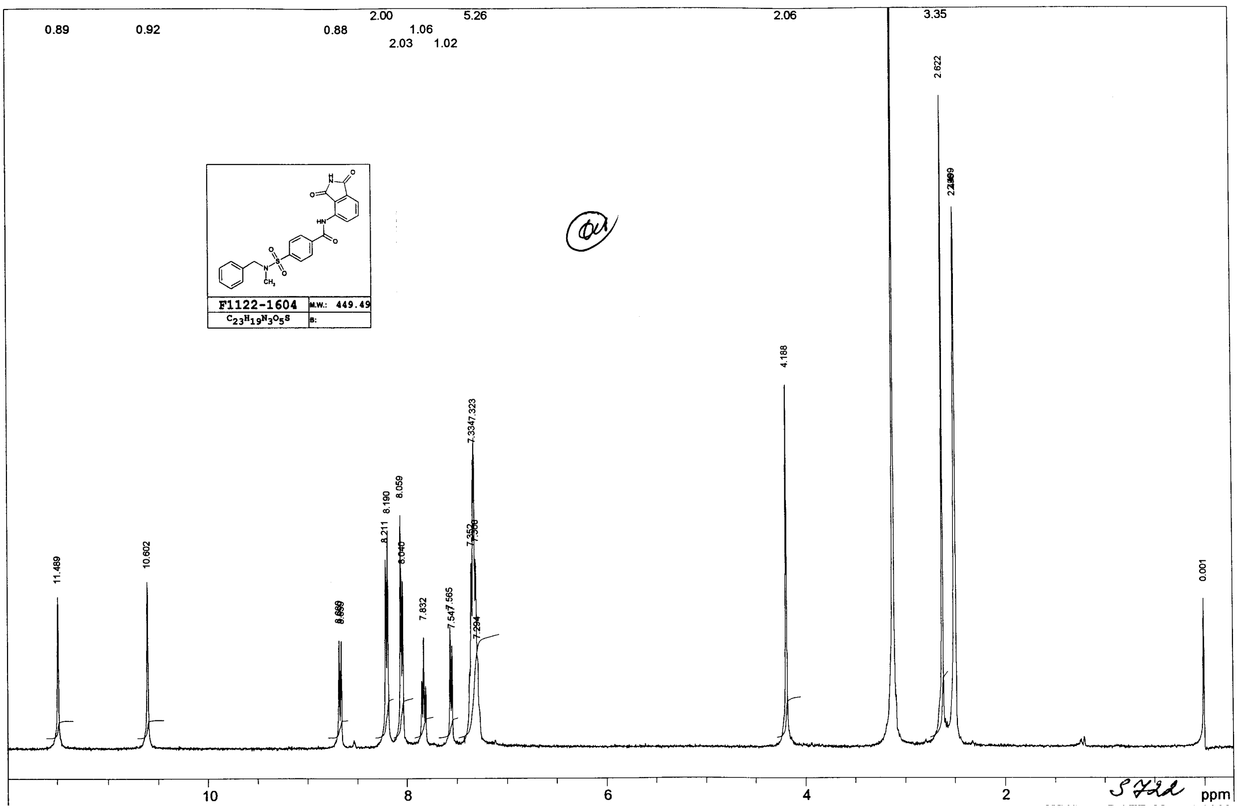

Compound 07

(Life Chemicals. ID: F2728-0771)

^{1}H NMR Spectrum

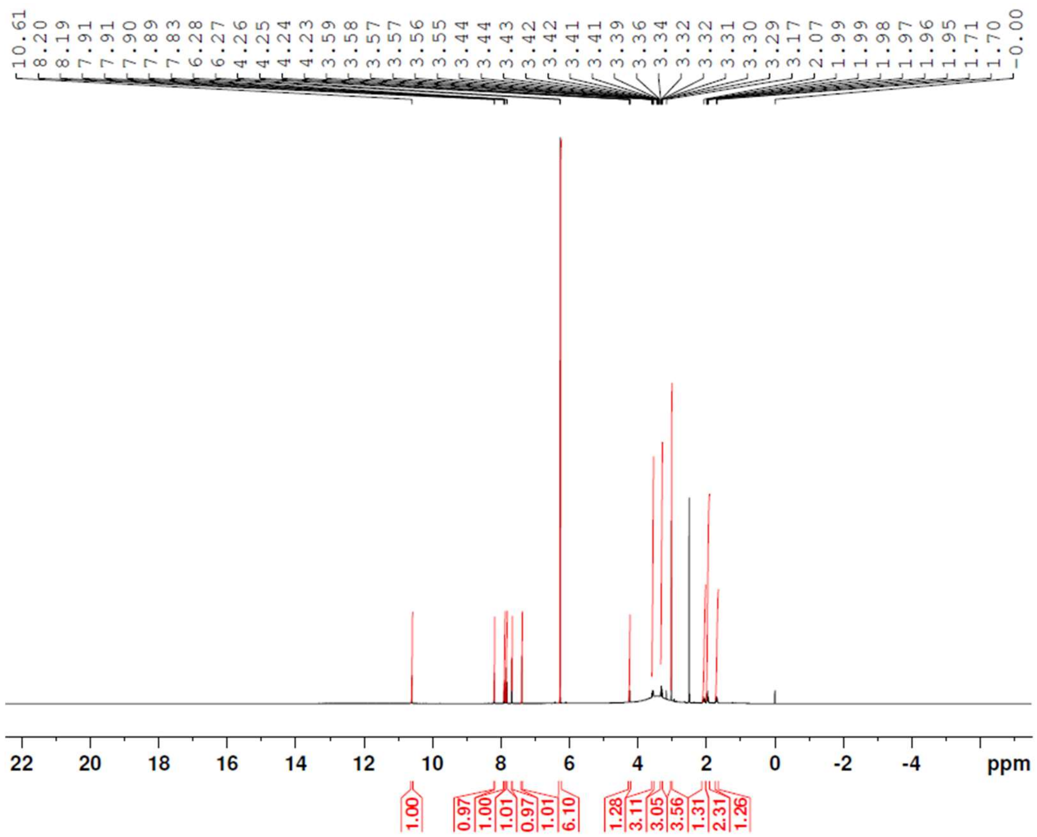

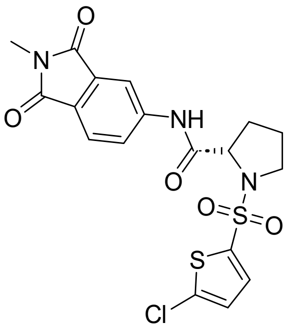

Compound 08

(Life Chemicals. ID: F3353-0159)

[1]*H NMR Spectrum* (provided by the supplier)

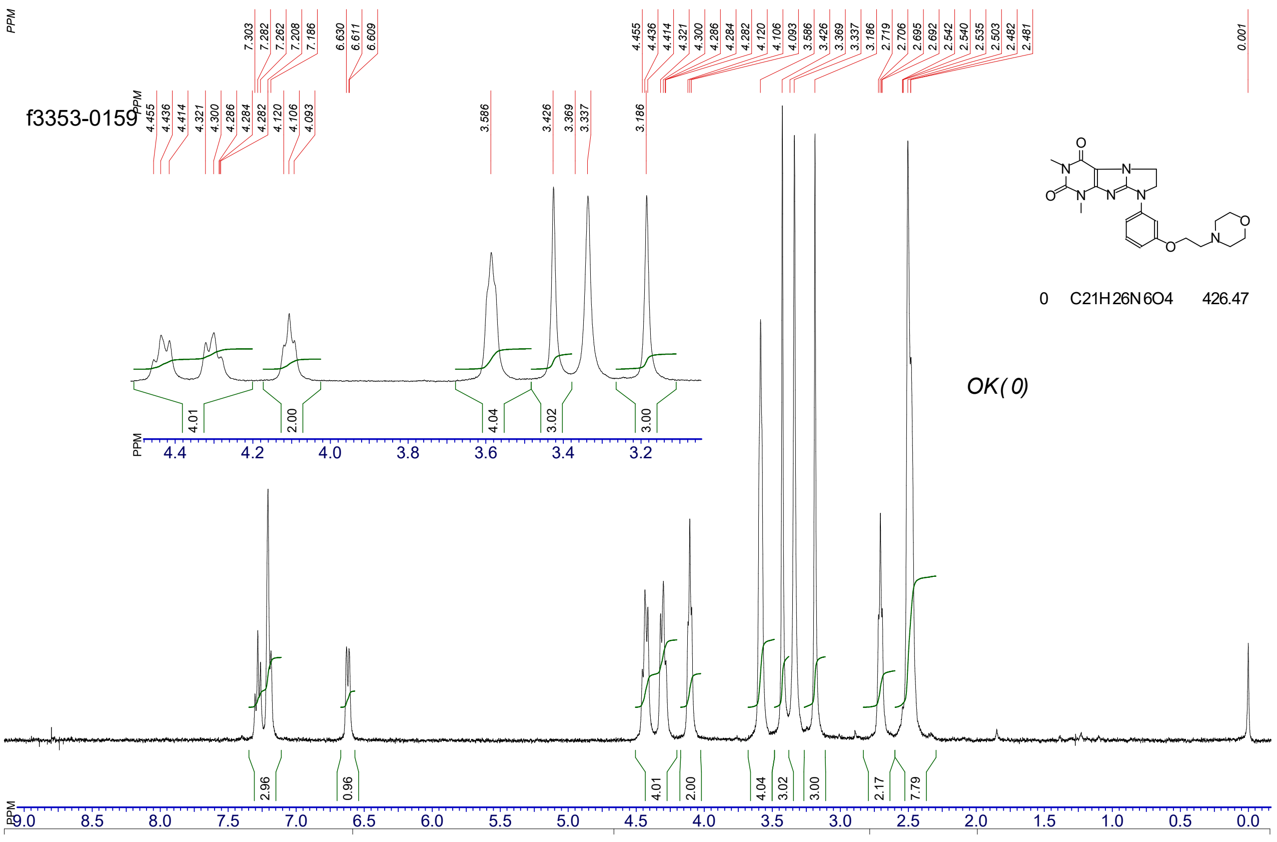

Compound 09

(Life Chemicals. ID: F5485-0702)

LC-MS Spectrum **(provided by the supplier)**

-.o.-Syntez Purity Report -.o.-

Agilent 1100 LC/MSD SL
Diodearrey G1315B (DAD1A-215nm; DAD1B-254nm)
Mass Quad G1956B (MSD1-Pos, MSD2-Neg)
ELSD Altech 3300 (ADC1 A, ELSD)

Separation column:
Rapid Resolutionn HT Cartige 4.6x30mm,
1.8-Micron, Zorbx SB-C18

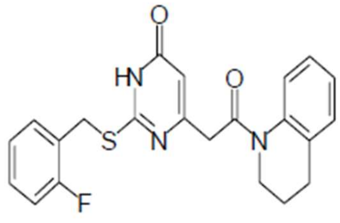

Mol.Weight: 409,49

F5485-0702

M3240 ->

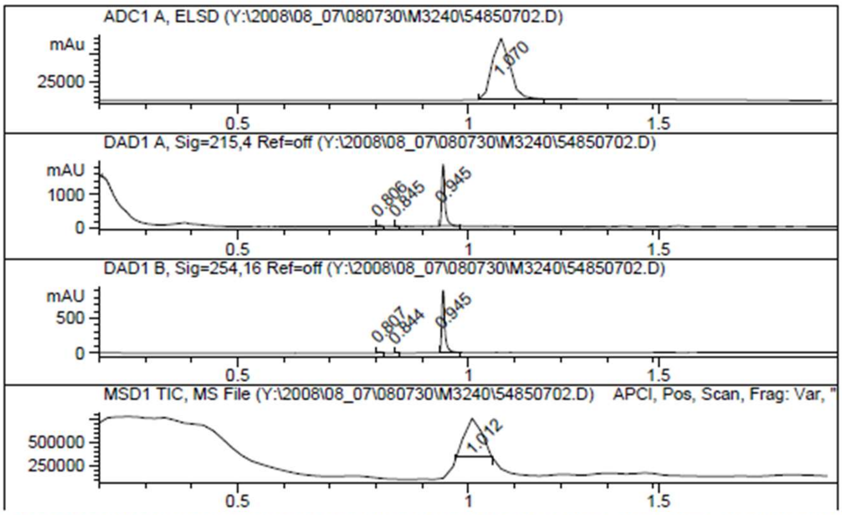

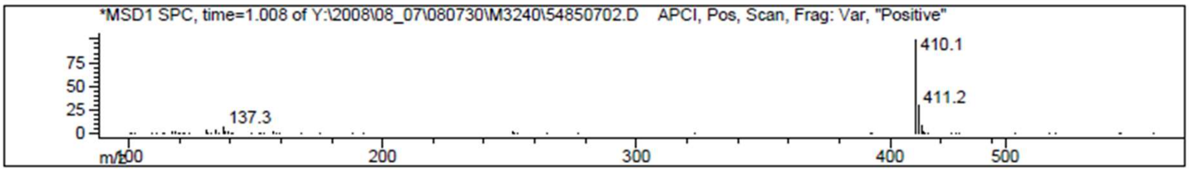

#	Signal	R.Time	Area %
1	ADC1 A, ELSD	1.070	100.000

#	Signal	R.Time	Area %
1	DAD1 A, Sig=215,4 Ref=off	0.806	2.148
2		0.845	0.918
3		0.945	96.934

#	Signal	R.Time	Area %
1	DAD1 B, Sig=254,16 Ref=off	0.807	2.247
2		0.844	0.778
3		0.945	96.974

#	Signal	R.Time	Area %
1	MSD1 TIC, MS File	1.012	100.000

Compound 10

(Vitas-M Laboratory. ID: STK705826)

^{1}H NMR Spectrum

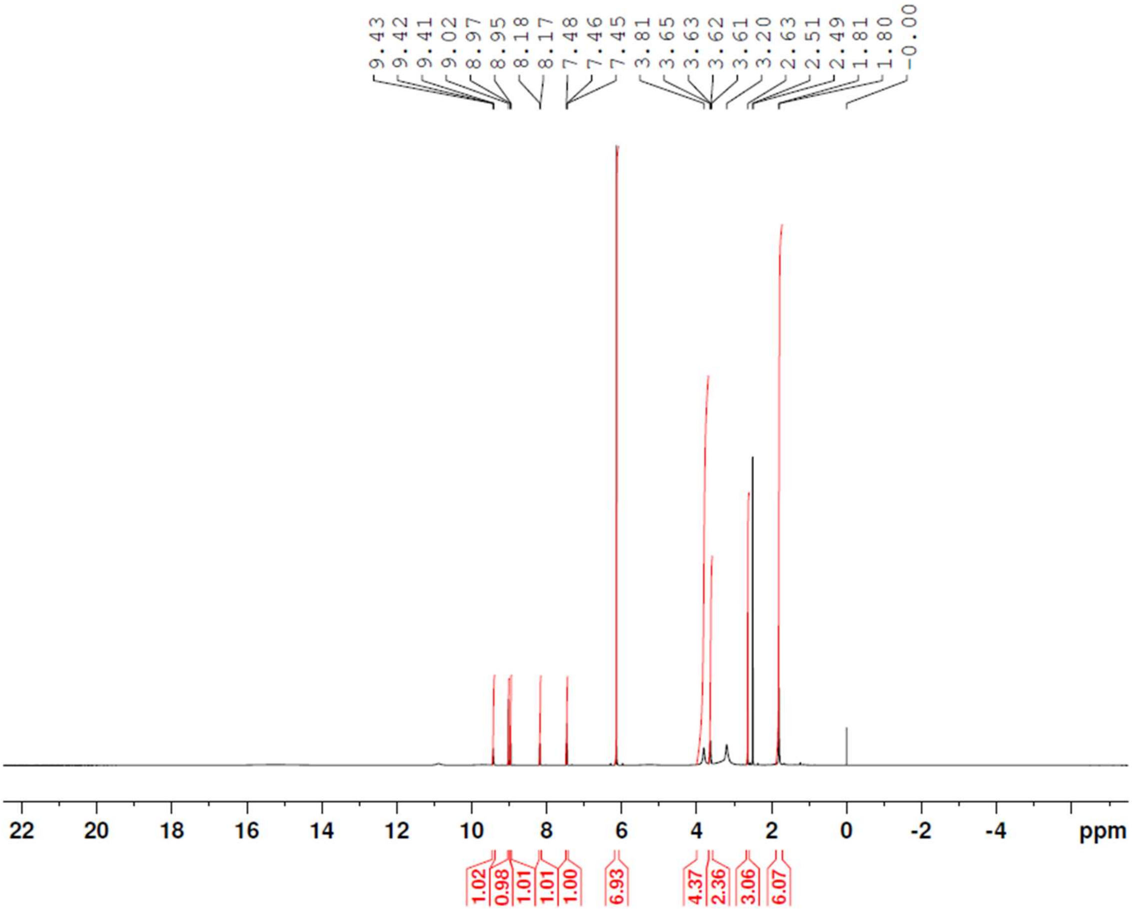

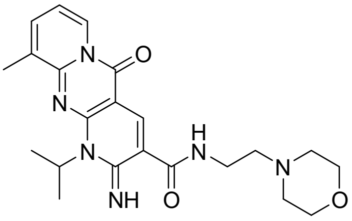

Compound 11

(Vitas-M Laboratory. ID: STK801051)

^{1}H NMR Spectrum

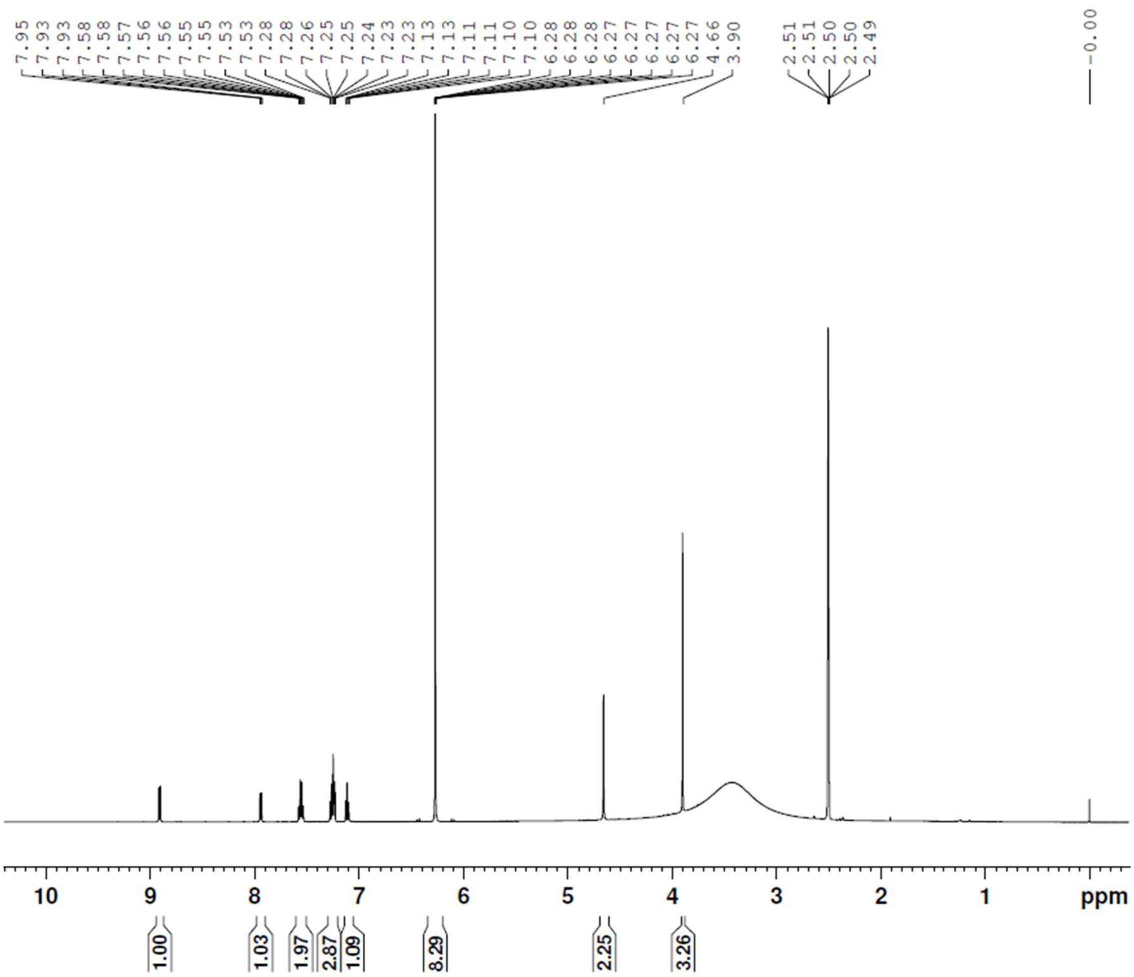

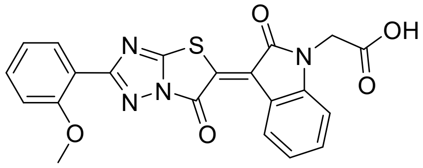

Compound 12

(Vitas-M Laboratory. ID: STL537989)

^{1}H NMR Spectrum

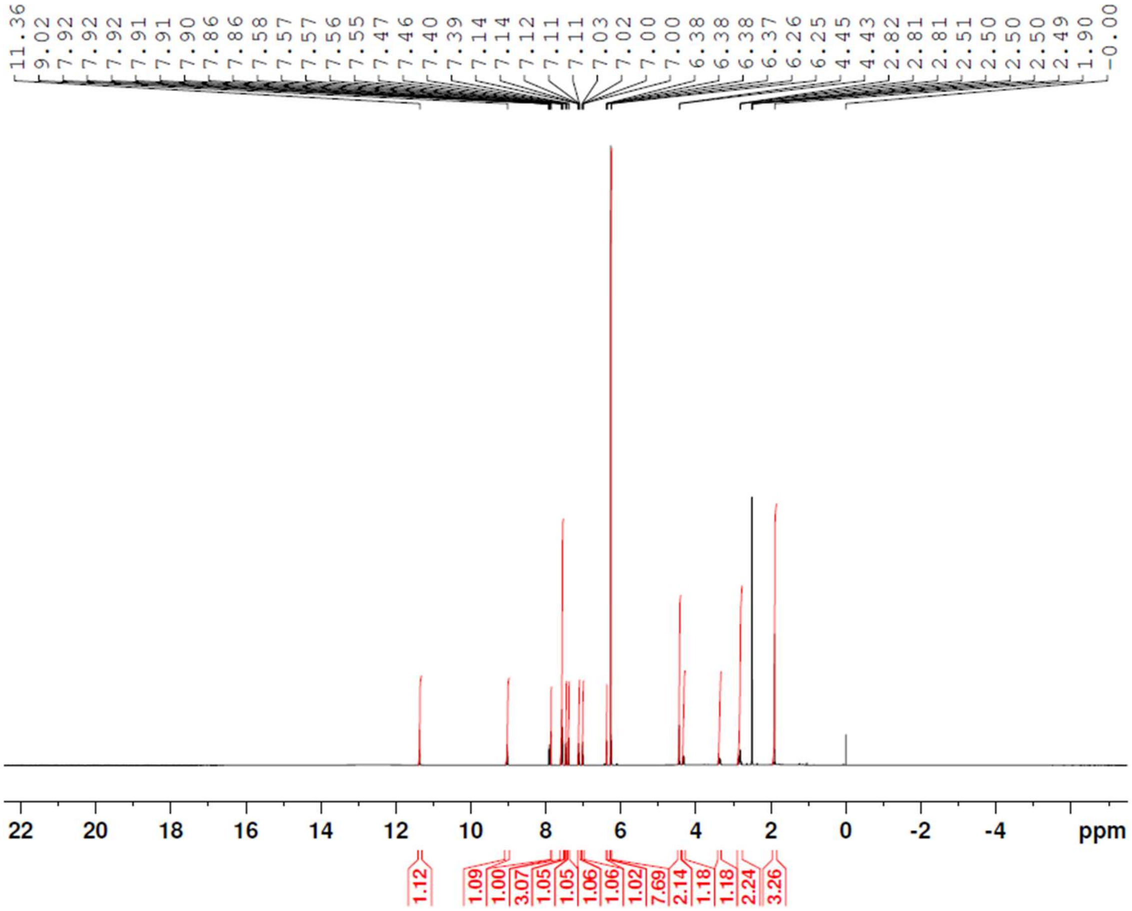

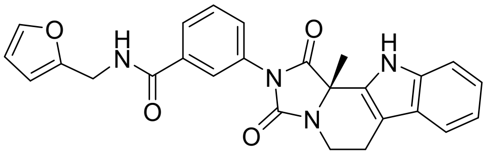

Compound 13

(Vitas-M Laboratory. ID: STK530237)

^{1}H NMR Spectrum

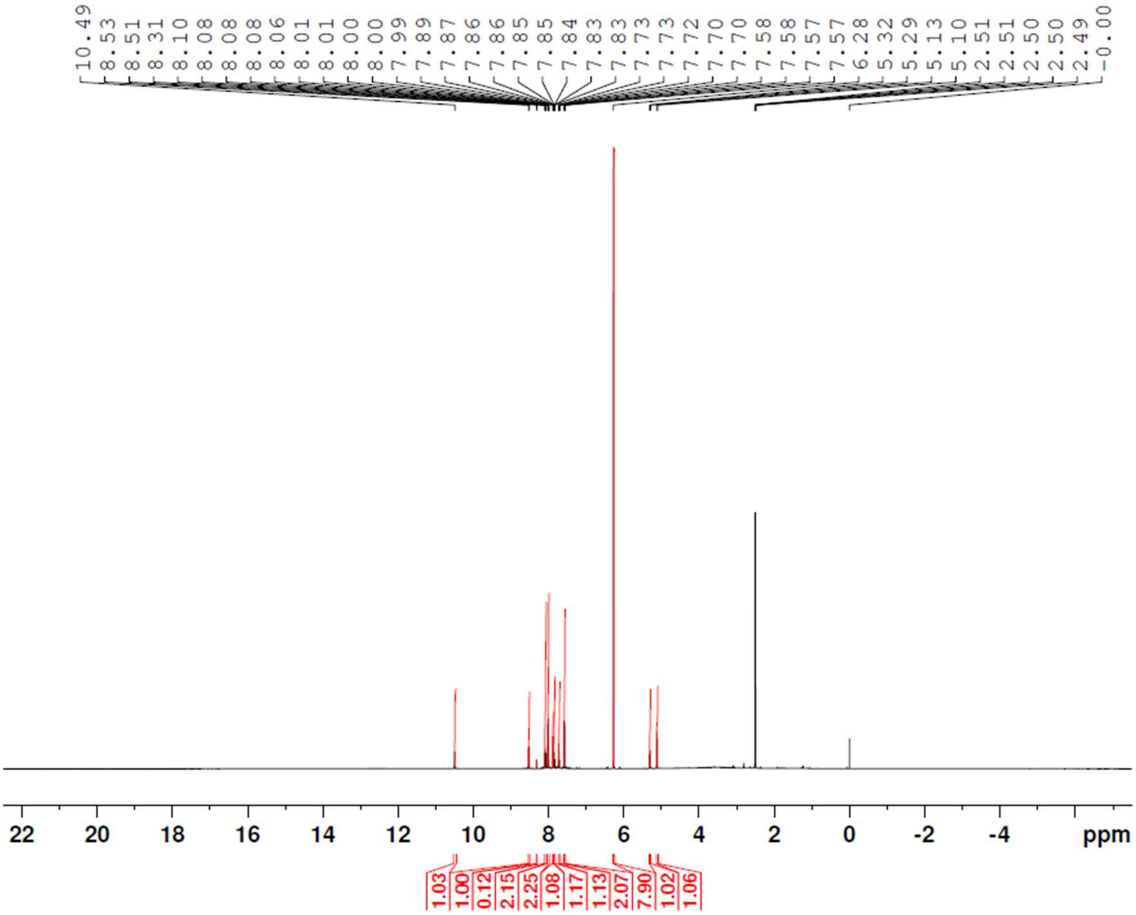

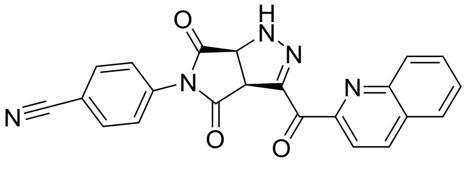

Compound 14

(Vitas-M Laboratory. ID: STL519482)

^{1}H NMR Spectrum

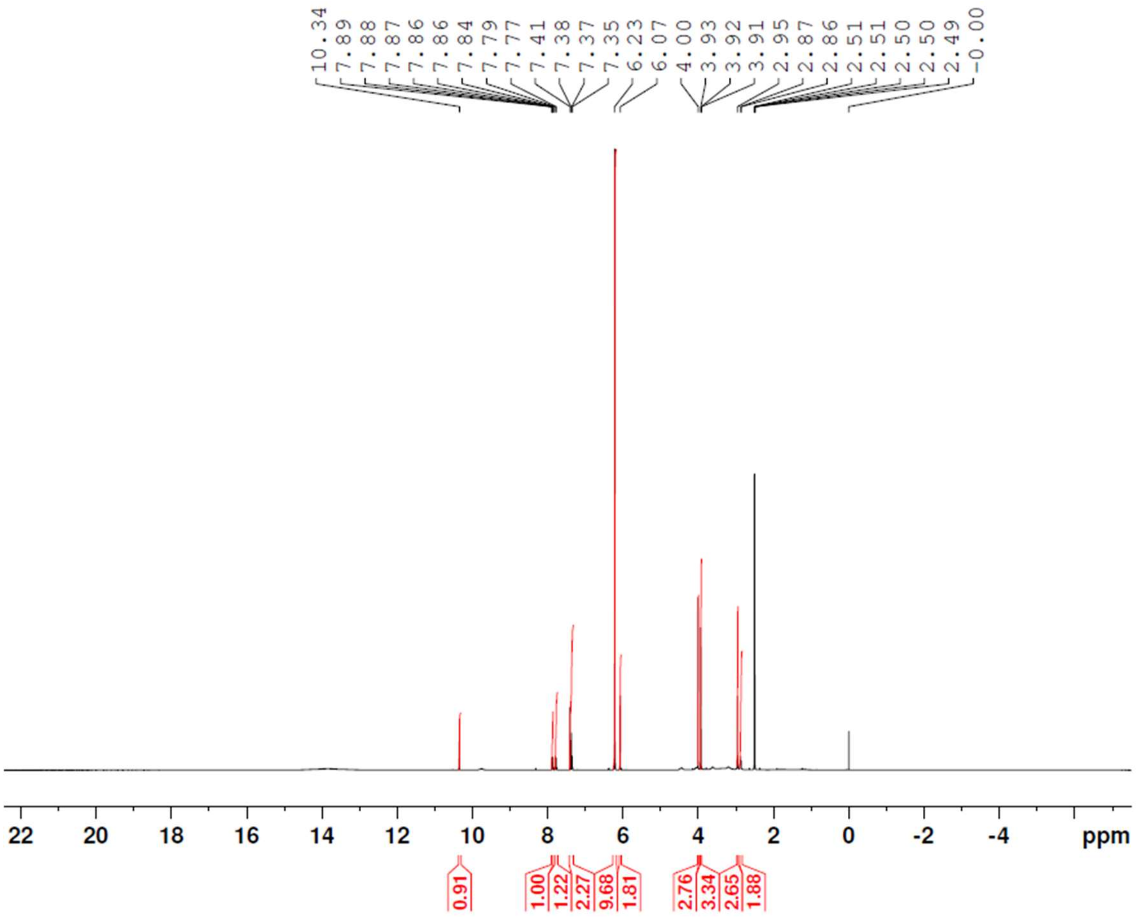

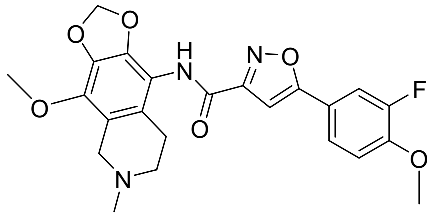

Table S9. Data used to calculate the purities of compounds **07**, **10**, **11**, **12**, **13**, and **14**.[(a)]

	Compound ID					
	**07**	**10**	**11**	**12[(b)]**	**13**	**14**
Number of protons corresponding to integrated peak(s) (sample), n_s	4	6	2	-	10	3
Number of protons corresponding to integrated peak (internal calibrant), n_{IC}	2	2	2	-	2	2
Sum of integrals of peaks from the sample, Int_s	3.9917	6.0661	2.2515	-	10.8438	2.7566
Integral of peak from the internal calibrant, Int_{IC}	6.095	6.9257	8.2855	-	7.9016	9.6813
Molecular weight (sample), MW_s (g.mol^{-1})	453.9	425.5	433.4	-	395.4	456.5
Molecular weight (internal calibrant) (g.mol^{-1}), MW_{IC}	116.07	116.07	116.07	-	116.07	116.07
Mass (sample), m_s (mg)	1.52	1.49	1.098	-	1.28	1.1
Mass (internal calibrant), m_{IC} (mg)	1.05	1.45	0.957	-	1.22	1.33
Purity (internal calibrant), P_{IC} (%)	99.98	99.98	99.98	-	99.98	99.98
**Purity (sample)**	**88.44**	**104.14**	**88.42**	**-**	**89.10**	**90.25**
Chemical shift(s) of the sample used for calculation (ppm)	7.91<br>7.84<br>7.68<br>7.40	1.80	4.65	-	8.52<br>8.09<br>8.01<br>7.88<br>7.84<br>7.72<br>7.57	4.00
Chemical shift(s) of the internal calibrant used for calculation (ppm)	6.13	6.27	6.27	-	6.27	6.22

[(a)] Calculated using the following equation:

$$Purity\ (sample) = \frac{n_{IC}.Int_s.MW_s.m_{IC}}{n_s.Int_{IC}.MW_{IC}.m_s}.P_{IC}$$

[(b)] Purity value for compound **12** could not be accurately calculated due to superposition of signals of the sample with the signal of the internal calibrant.

References

(1) Tsai, W. C.; Gilbert, N. C.; Ohler, A.; Armstrong, M.; Perry, S.; alyanaraman, C.; Yasgar, A.; Rai, G.; Simeonov, A.; Jadhav, A.; Standley, M.; Lee, H. W.; Crews, P.; Iavarone, A. T.; Jacobson, M. P.; Neau, D. B.; Offenbacher, A. R.; Newcomer, M.; Holman, T. R. Kinetic and Structural Investigations of Novel Inhibitors of Human Epithelial 15-Lipoxygenase-2. *Bioorg Med Chem* **2021**, 46. https://doi.org/10.1016/j.bmc.2021.116349.

(2) Kobe, M. J.; Neau, D. B.; Mitchell, C. E.; Bartlett, S. G.; Newcomer, M. E. The Structure of Human 15-Lipoxygenase-2 with a Substrate Mimic. *J Biol Chem* **2014**, 289 (12), 8562–8569. https://doi.org/10.1074/jbc.M113.543777.

(3) Daina, A.; Michielin, O.; Zoete, V. SwissADME: A Free Web Tool to Evaluate Pharmacokinetics, Drug-Likeness and Medicinal Chemistry Friendliness of Small Molecules. *Sci Rep* **2017**, 7. https://doi.org/10.1038/srep42717.

(4) Buker, S. M.; Boriack-Sjodin, P. A.; Copeland, R. A. Enzyme–Inhibitor Interactions and a Simple, Rapid Method for Determining Inhibition Modality. *SLAS Discovery* **2019**, 24 (5), 515–522. https://doi.org/10.1177/2472555219829898
